# An integrated transcriptomic and metabolomic approach to investigate the heterogeneous *Candida albicans* biofilm phenotype

**DOI:** 10.1016/j.bioflm.2023.100112

**Published:** 2023-03-12

**Authors:** Christopher Delaney, Bryn Short, Ranjith Rajendran, Ryan Kean, Karl Burgess, Craig Williams, Carol A. Munro, Gordon Ramage

**Affiliations:** a School of Medicine, College of Medical, Veterinary and Life Sciences (MVLS), University of Glasgow, UK; b Department of Biological and Biomedical Sciences, School of Health and Life Sciences, Glasgow Caledonian University, Glasgow, UK; c Institute of Quantitative Biology, Biochemistry and Biotechnology, University of Edinburgh, Max Born Crescent, Edinburgh, UK; d Microbiology Department, Lancaster Royal Infirmary, University of Lancaster, Lancaster, UK; e School of Medicine, Medical Sciences and Nutrition, Institute of Medical Sciences, University of Aberdeen, UK

**Keywords:** *Candida albicans*, Biofilm, Transcriptomics, Metabolomics

## Abstract

*Candida albicans* is the most prevalent and notorious of the *Candida* species involved in bloodstream infections, which is characterised by its capacity to form robust biofilms. Biofilm formation is an important clinical entity shown to be highly variable among clinical isolates. There are various environmental and physiological factors, including nutrient availability which influence the phenotype of *Candida* species. However, mechanisms underpinning adaptive biofilm heterogeneity have not yet been fully explored.

Within this study we have profiled previously characterised and phenotypically distinct *C. albicans* bloodstream isolates. We assessed the dynamic susceptibility of these differing populations to antifungal treatments using population analysis profiling in addition to assessing biofilm formation and morphological changes. High throughput methodologies of RNA-Seq and LC-MS were employed to map and integrate the transcriptional and metabolic reprogramming undertaken by heterogenous *C. albicans* isolates in response to biofilm and hyphal inducing serum.

We found a significant relationship between biofilm heterogeneity and azole resistance (P < 0.05). In addition, we observed that in response to serum our low biofilm forming (LBF) *C. albicans* exhibited a significant increase in biofilm formation and hyphal elongation. The transcriptional reprogramming of LBF strains compared to high biofilm forming (HBF) was distinct, indicating a high level of plasticity and variation in stress responses by heterogenous strains. The metabolic responses, although variable between LBF and HBF, shared many of the same responses to serum. Notably, a high upregulation of the arachidonic acid cascade, part of the COX pathway, was observed and this pathway was found to induce biofilm formation in LBF 3-fold.

C. albicans is a highly heterogenous bloodstream pathogen with clinical isolates varying in antifungal tolerance and biofilm formation. In addition to this, *C. albicans* is capable of highly complex and variable regulation of transcription and metabolic pathways and heterogeneity across isolates further increases the complexity of these pathways. Here we have shown with a dual and integrated approach, the importance of studying a diverse panel of *C. albicans* isolates, which has the potential to reveal distinct pathways that can harnessed for drug discovery.

## Highlights

• *Candida albicans* clinical isolates display a heterogeneous phenotype
• Heterogeneity correlates with azole tolerance
• Heterogeneous clinical isolates display adaptable metabolic plasticity
• Arachidonic acid liberated from host factors induces biofilm formation and is therefore a putative therapeutic target.

## 1 Introduction

*Candida albicans* is the most prevalent and pathogenic of the *Candida* species involved in bloodstream infections [1], and is on the World Health Organisation's list of critical priority fungal pathogens. We have previously reported that *C. albicans* was found in 41% of patients from a Scottish cohort of 217 patients, followed by *C. glabrata* (35%). Notably, despite the absence of fluconazole (FLU) resistance in patients receiving azole therapy, we reported a 41% mortality rate. *C. albicans* is typically characterised by its capacity to form differentially adherent biofilms on the luminal surface of catheters, thus facilitating entry to the bloodstream. Biofilms are an important clinical entity and are highly variable among clinical isolates [1,2]. Fungal biofilms are remarkably difficult to treat with antifungal agents, and indeed our studies have shown a positive correlation between biofilm formation and mortality [3,4]. Moreover, these data point towards the importance of strain heterogeneity and the possibility that strain phenotype and fitness are important determinants of clinical outcomes.

Our original assumptions suggested that constitutive biofilm formation phenotypes were a key determinant of mortality, but we also observed strains with lower biofilm forming capacity had a propensity to be induced into a biofilm phenotype. There are various environmental and physiological factors, including nutrient availability, hypoxia, pH and temperature that influence the lifestyle of *Candida* species [[5], [6], [7]]. The interaction of *Candida* with serum has been of long-standing concern in the aetiology of fungal bloodstream infections. Serum has been shown to be a promoter of adhesion and filamentation in *C. albicans,* potentially influencing the biofilm phenotype [[8], [9], [10]]. Indeed, it has been demonstrated that human serum stimulates *C. albicans* biofilm formation on silicone discs [11]. Certain *in vivo* environments, such as in presence of serum, are thought to induce amino acid starvation conditions, genes for the biosynthesis and metabolism of various amino acids and the general regulator Gcn4p are induced upon exposure of *Candida* to blood [[12], [13], [14]]. Gcn4p is a major transcriptional activator that regulates over 30 genes for biosynthesis of 19 amino acids, as well as controlling many other cellular processes, including purine biosynthesis, organelle biosynthesis, and stress responses [15]. Additionally, the presence of serum is thought to upregulate biofilm formation through activation of Ras1. Ras1 interacts with Cyr1 which in turn activates the cAMP-PKA pathway [16,17]. However, there is scant information about the role played by serum in *C. albicans* biofilm heterogeneity amongst wild type clinical strains. We reported the role of different metabolic pathways, including amino acid metabolism in *C. albicans* biofilm strains expressing an optimal biofilm phenotype [18]. However, the mechanisms underpinning adaptive biofilm heterogeneity in strains with a less pronounced biofilm phenotype have not yet been fully explored. Moreover, much of our understanding of *C. albicans* adaptation to host environment have to date been garnered from studies of planktonic cultures [19].

The control of cellular physiology in response to immediate environmental changes involves reprogramming of gene expression regulatory systems as well as the post-translational modulation of metabolic pathways [9]. Metabolic adaptation facilitates *C. albicans* to assimilate nutrients and grow in a variety of host environments [6]. A key component of such adaptation is filamentous growth, which is stimulated by a variety of factors, including carbohydrate source and amino acid starvation [[5], [6], [7]]. Changes in metabolic pathways impact *C. albicans* pathogenicity, which includes yeast-hypha morphogenesis, phenotypic switching, and expression of adhesins, invasins, and secreted hydrolases [6], factors that are important for the biofilm phenotype [20]. In fact, these biofilm related processes are driven by 6 key transcriptional regulators as part of a complex transcriptional circuitry [21]. While this level of global control is well understood, how these relate to central metabolic processes is limited. Although several anabolic and catabolic pathways, such as those involved in carbon and amino acid metabolism, have been shown to play a pivotal role in *C. albicans* biofilm formation, the key pathways associated with an adaptive biofilm phenotype remain unclear and have not yet been explicitly examined [22,23].

In this current study, we evaluated the hypothesis that metabolic adaptation plays an important role in *C. albicans* biofilm adaptation. This project aimed to dissect the role of adaptive metabolism in the formation of *C. albicans* biofilms from strains that typically form scant, incoherent biofilm structures using a combination of transcriptomic and metabolomic analyses. For the first time we report an integrated analysis of the transcriptomic and metabolomic adaptation of *C. albicans* clinical isolates and demonstrate the importance of arachidonic acid cascade inducing a biofilm phenotype.

## 2 Materials and methods

### 2.1 Culture conditions and standardisation

This study utilised clinical *Candida albicans* (n = 60) bloodstream isolates, collected under the approval of the NHS Scotland Caldicott Gaurdian's [3,24]. Isolates were stored in Microbank® vials (Pro-Lab Diagnostics, Cheshire, UK) at −80 °C until sub-cultured onto Sabouraud's dextrose agar (SAB [Sigma-Aldrich, Dorset, UK]). *C. albicans* isolates were propagated in yeast peptone dextrose (YPD) medium (Sigma-Aldrich, Dorset, UK), washed by centrifugation and re-suspended in RPMI-1640 with 2% D-glucose (RPMI-1640, Sigma-Aldrich, Dorset, UK) to 1 × 10^6^ cells/mL, as described previously [19].

### 2.2 Strain characterisation

In order to assess and stratify the phenotypes of the clinical isolates we undertook basic biofilm growth characterisation and population analysis profiling (PAP). Initially, *C. albicans* biofilms were grown according to our established protocols [3]. Pre-characterised *C. albicans* isolates with HBF or LBF biofilm forming ability were used throughout this study [3,24]. For all experiments, biofilms were grown in polystyrene plates or 75 cm^2^ tissue culture flasks or Nunc™ Thermanox™ coverslips in RPMI-1640 (Sigma) for 90min, 4 or 24 h at 37 °C. For induction experiments, RPMI media was supplemented with foetal calf serum (FCS [25% (v/v)]) or dialysed serum (DS [25% (v/v)]). Serum was dialysed using a Spectra/Por™ (fisher scientific) dialysis membraned with a 3.5kD molecular weight cut-off to remove small molecules. The biofilm biomass of each isolate was assessed with the crystal violet (CV) assay as previously described [3]. Hyphal length formation was quantified using a light microscope and eye piece graticule and stage micrometre for HBF (n = 1, [39]) and LBF (n = 1, [204]) clinical isolates.

As a secondary method of stratifying the isolates PAP was used to assess tolerance to different antifungals [25]. The aim of these experiments was to investigate the dynamic response to different antifungals drugs from clinical isolates that appeared to have similar MICs as judged by CLSI endpoint assay determinations, and to further correlate this with biofilm growth. We analysed the population distribution of *C. albicans* isolates for susceptibility to fluconazole (FLZ), caspofungin (CAS), and amphotericin B (AMB) by this spectrophotometric method. In brief, standardised 10^4^ cells/mL were treated with different concentrations of FLZ (0–128 mg/L), CAS (0–8 mg/L) and AMB (0–8 mg/L) in RPMI-1640, at 37 °C for 24h. Although this assay is similar to MIC testing, the purpose was not to determine the endpoint MIC concentration; instead, we calculated the tolerance range (TR) from the minimum concentration at which the cell growth started reducing to the concentration where it reaches the base level growth. After incubation period, wells were mixed thoroughly and absorbance measured at a wavelength of 530 nm. Then colony forming units (CFU) per mL was calculated using a standard curve. For each isolate, the CFU value was plotted over the drug concentration. To quantitate tolerance, we calculated the area under the population distribution curve (AUC). Pairwise correlation between biofilm formation and AUC were found by calculating 2-tailed Pearson correlation coefficient. All the graphs were generated in R with the use of ggplot2 package.

Finally, to determine the importance of the Arachidonic Acid (AA) cascade in *C. albicans* biofilm formation, *in vitro* biofilm testing was performed by exposing individual 24 h biofilms formed by HBF (n = 5, [31,39,48G,177A,198]) and LBF (n = 5, [17B, 88C, 89B, 140, 204]) *C. albicans* isolates to AA (Sigma-Aldrich) in the presence and absence of the COX pathway inhibitor, salicylic acid (Sigma-Aldrich). Biofilms were exposed to AA and salicylic acid, which were diluted to 5 mM in RPMI-1640, for a further 24 h. Appropriate, untreated biofilm controls were included which received sterile RPMI-1640 medium. Following biofilm treatments, biofilms were washed with PBS to removed non-adherent cells and changes in biofilm biomass were quantified via crystal violet staining where the absorbance of bound dye was measured at 570 nm [19]. GraphPad Prism (version 9; La Jolla, California) was used to visualise and analyse crystal violet absorbance data. These data were analysed using the Kruskal-Wallis test followed by Dunn's test to correct for multiple comparisons.

### 2.3 Electron microscopy

HBF (n = 1, [39]) and LBF (n = 1, [204]) isolates were grown directly onto Thermanox™ coverslips (Nunc, Roskilde, Denmark) for 24h in RPMI-1640 the presence and absence of 25% FCS. After incubation period, biofilms were carefully washed with PBS and processed for Scanning Electron microscopy (SEM) as previously described [26]. Briefly, samples were fixed in 2% paraformaldehyde, 2% glutaraldehyde, and 0.15% [wt/vol] alcian blue in 0.15 M sodium cacodylate (pH 7.4). The biofilms were sputter coated with gold and viewed under a JEOL JSM-6400 scanning electron microscope.

### 2.4 RNA extraction and sequencing

HBF (n = 1, [39]) and LBF (n = 1, [204]), candidemia isolates, were washed with PBS before a cell scraper was used to dislodge the biomass, which was homogenised using a bead beater, and RNA extracted using the TRIzol™ (Life Technologies, Paisley, UK) method as described previously [27]. Total RNA was DNase (Qiagen, Crawley, UK) treated and purified using an RNeasy MiniElute clean up kit (Qiagen, Crawley, UK), as per manufacturer's instructions. RNA was quantified and quality assessed using a NanoDrop spectrophotometer (ND-1000, ThermoScientific, Loughborough, UK). Each isolate was grown in triplicate and a minimum of 10 μg of total RNA was submitted for each sample and sent for sequencing to The GenePool (Edinburgh, UK). RNA integrity was assessed using a Bioanalyzer where an RIN value > 7.0 was deemed acceptable for RNA-Seq using Illumina 50 base sequencing.

### 2.5 RNA-seq analysis

RNA-seq reads were processed by first quality controlled using the software Trimmomatic v0.38 [28] to remove Illumina adapters low quality bases leading = 3 and trailing = 3 and reads with remaining length of less than 30 bases. An index of the *Candida* genome (CGD) database reference *C. albicans* genome (SC5313_A22) was then constructed using Hisat2 [29]. The current genome maintained by the Candida Genome Database (CGD) is diploid, we utilised a haploid genome for RNA-Seq analysis by disregarding the B variants of the chromosomes from the fasta and gff annotation files.

Hisat2 (v2.1.0) was then utilised to map the trimmed sample reads to the SC5314 genome [29]. Subsequent SAM files containing the aligned reads were then coordinate sorted and converted to BAM format using Samtools (v1.7) [30]. Quality of the alignments was assessed using the software Qualimap (v.2.2.2) and the BAM files for each sample were counted to obtain gene counts using the corresponding SC5314_A22 gff file from the *Candida* genome database with the use of the program HTSeq-count (0.11.0) [31,32]. All gene counts were then parsed into a single large array containing all the of the samples and the corresponding counts for each of the genes. The gene count array was then analysed within the R programming environment assisted by the R Studio GUI (http://www.rstudio.com/). Differential expression analysis was largely performed with the assistance of the DESeq2 (v1.26) R package [33]. DESeq2 uses a negative binomial model to estimate gene abundance and differentia expression between variables. Differential expression was performed in a pairwise fashion between sample variables and significance was determined if genes had a Log2FC > 1.5 and an FDR adjusted p-value of <0.05. *C. albicans* transcripts that were significantly differentially expressed between pairwise comparisons underwent further functional analysis within the network software Cytoscape (v3.7.2) [34]. The plugin GlueGO annotated and grouped the genes into functional categories and significantly over-represented categories were found using the hypergeometric test which deemed functional categories to be enriched with an adjusted p-value <0.05. Networks of over-represented functional categories using either the gene ontology or the KEGG databases were constructed using GlueGO (v2.5.5) plugin and drawn within the Cytoscape environment [35]. Additionally, fgsea (v1.12.0) was used to perform GSEA from within the clusterProfiler (v3.14.3) R package [36]. Multivariate analysis and important feature identification were performed through the mixOmics (v6.10.9) R package utilising their sPLS and PLS-DA functions [37].

### 2.6 Metabolomics

The LC-MS identification of metabolites was performed by the Glasgow Polyomics MS facility based upon there previously validated protocols [38,39]. Briefly, HBF (n = 3, [39, 48G, 177A]) and LBF (n = 3, [17B, 140, 204]) isolates were grown as biofilms for 4 and 24 h in RPMI ± FCS as described above. 5 μL of the spent media was added to 200 μL of an ice cold solvent solution containing chloroform:methanol:water in a (1:3:1:v:v:v) ratio. This solution was then centrifuged at maximum speed and stored at −80 °C until being run on a pHILIC columns LC-MS analysis. Fresh media was used as a control and solvent solution was used as a blank. All strains were grown in triplicate for a total of 9 repeats. MS raw data files were filtered and converted to mzXML using msconvert component of the ProteoWizard software package (Chambers et al., 2012). mzXML files were submitted to the Polyomics integrated Metabolomics Pipeline (PiMP). Principal metabolites were identified against a panel of authentic pure standards (Sigma-Aldrich), that are maintained in the University of Glasgow Polyomics standard compound library (https://www.polyomics.gla.ac.uk/ms_metabolomics.html), using accurate mass and RT (error 5%) and were defined as Identified based upon the Metabolite Standards Initiative.

## 3 Results and discussion

### 3.1 *Candida albicans* biofilm formation correlates with azole tolerance

We demonstrated previously that biofilm heterogeneity (classified as either high [HBF] or low [LBF] biofilm formers is an important clinical trait for *C. albicans* isolates [3,24]. We had previously identified differences between the transcriptomic response of two clinical distinct isolates [18]. Notably, in these studies we found that LBF isolates were more adaptable in nature and formed biofilms in response to a defined nutrient stimulus, whereas HBF isolates exhibited a constitutive biofilm phenotype. We aimed to assess whether the strains isolated showed any addition differences in other phenotypic characteristics, such as antifungal tolerance and whether this correlated with biofilm attributes. This would provide reassurance of the distinct phenotypes.

**Fig. 1:**
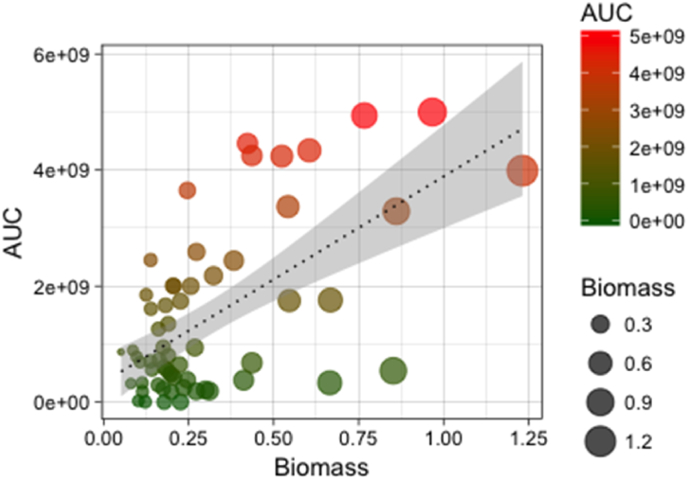
**Relationship between fluconazole tolerance and biofilm formation.** Correlation between tolerance (area under the curve [AUC]) and biofilm formation (biomass) is shown. Colour gradient from green to red indicates low to high tolerance to fluconazole and circle size indicates the level of biofilm formation (R = 0.34, p < 0.05).

We initially characterised the drug tolerance and biofilm forming ability of 60 *C. albicans* clinical isolates that were previously determined to be susceptible to FLU by standard broth microdilution methods [4]. Population analysis profiling (PAP) assays were analysed by determining FLU tolerance and calculating the AUC, which provides a continuous measurement of strain heterogeneity. FLU tolerance among all 60 *C. albicans* clinical isolates was found to be highly variable, with an AUC of 2–256 and AUC ranged from 0 to 5 × 10^9^. However, on stratification of biomass versus AUC, we observed a significant correlation between biofilm formation and FLU tolerance (Fig. 1). Whereas, for caspofungin and amphotericin B we observed that there was no significant correlation between biofilm forming ability and tolerance (Supplementary 1). Together these data suggest that biofilm forming capabilities are related to a delayed response to azole antifungal, though this does not fully translate to all classes of antifungals. We can therefore surmise that we could differentiate the clinical strains based on these two criteria, i.e. biofilm formation and a diminished azole tolerance. This is an important observation to highlight because despite these isolates appearing equally sensitive to azoles, in our clinical data we show a variable mortality outcome [3,4]. While we have reported a correlation between biofilm formation and this *in vitro* biofilm phenotype, it is plausible that clinical outcomes may relate closely to a subtle azole tolerant phenotype that was not observable by standard methods. Indeed, it has been reported that the tolerance phenotype was predictive of how effective fluconazole can be if started early [40]. It is possible that these phenotypes may have some relevance to the heteroresistance phenotypes recently described in *C. glabrata* and in polymicrobial infection [25,41]. Together, these data imply the importance of discovering the molecular differences between these isolates.

### 3.2 Serum induces the adaptive biofilm phenotype

**Fig. 2:**
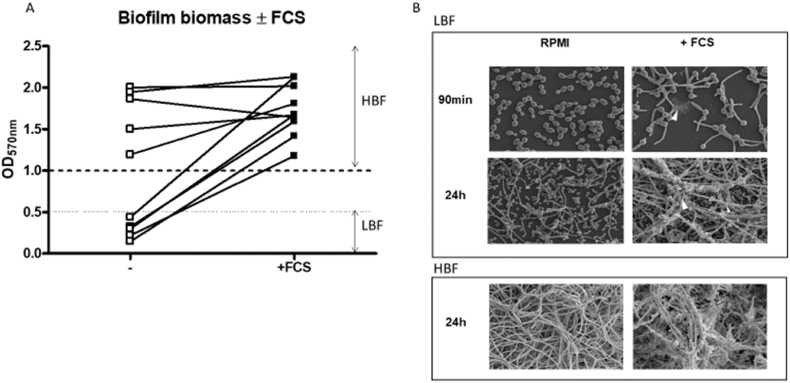
**Variation in *C. albicans* biofilm formation in the presence and absence of FCS.** Biofilm formation after 24h assessed using crystal violet assay of 5 low biofilm forming (LBF) and 5 high biofilm forming (HBF) isolates grown in RPMI or RPMI supplemented with serum **(A)**. Scanning electron microscopy (SEM) images of high biofilm forming (HBF) or low biofilm forming (LBF) isolates morphology grown similarly in the presence and absence of serum grown on coverslips for 90min or 24h **(B)**.

When *C. albicans* enters the bloodstream it is exposed to a range of host factors, but fundamentally it becomes stressed and undergoes morphological changes [42]. How this environment impacts LBF isolates and the activation of biofilm related metabolic pathways have not yet been evaluated. Therefore, we assessed biofilm induction by LBF isolates in the presence of FCS (LBF + FCS) or in its absence. Addition of FCS significantly induced biofilm formation by 5–7.4 times (p < 0.01) compared to isolates grown in RPMI control with no FCS added (Fig. 2). SEM images showed a striking difference in biofilm phenotype of LBF in presence and absence of FCS (Fig. 2B). At 90 min LBF + FCS samples had typically more hyphal cells with some extracellular matrix, compared to predominantly yeast cells in control LBF samples. At the 24h matured biofilm phase, LBF + FCS formed multi-layered hyphal structures glued together with extracellular matrix (Fig. 2B).

**Fig. 3:**
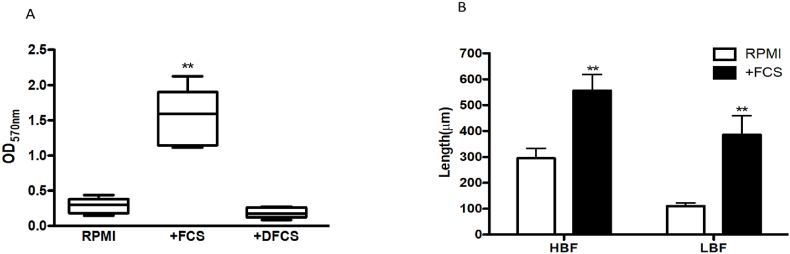
**Biomass of LBF isolates grown in RPMI and RPMI supplemented with 25% serum.** The effect of dialysation on the serum prior to supplementation was tested with biofilms grown in RPMI, RPMI supplemented with 25% serum or 25% dialysed serum for 24h. Significance was measured comparing RPMI control to RPMI supplemented with serum or dialysed serum by ANOVA with multiple comparison test showing significant differences (p < 0.01**) between RPMI and serum **(A)**. Bar chart depicting the overall length of hyphae in LBF and HBF candida isolates in the media RPMI and RPMI supplemented with 25% serum after 90min. Germ tube length measurements were taken and compared between groups using ANOVA with multiple comparison test displaying significant differences between germ tube length in RPMI compared to RPMI supplemented with serum in HBF and LBF (p < 0.01**) **(B)**.

Serum is a complex medium consisting of protein, lipids and small molecules. We therefore dialysed serum to determine whether the smaller molecules, including amino acids, played a role in biofilm induction. We found no significant biofilm up-regulation in the presence of dialysed serum (DS) (Fig. 3A [p > 0.05]), suggesting that small molecules were critical to biofilm induction. LBF biofilm architecture was scant, containing yeast and pseudohyphal cells. In addition, germ tube lengths of LBF and HBF isolates were significantly increased by 3.5- and 1.8-fold respectively, after 90 min of FCS exposure compared to RPMI only controls (Fig. 3B). These data indicate that the complex dialysable components of serum are responsible for induction of the biofilm phenotype and hyphal formation, particularly in the LBF. The components of serum are complex and signalling is potentially triggered by a number of pathways. Because of the numerous effectors of hyphal elongation and biofilm formation we also predict that this could vary between distinct clinical isolates. To identify these mechanisms it is necessary to adopt an integrated approach profiling transcriptional and metabolic changes by this environmental stimulant.

### 3.3 Transcriptional analysis reveals the role of transport and carbon metabolism in the induced biofilm phenotype

Next, to help understand how LBF strains adapted to the induced biofilm phenotype at a transcriptional level, RNA-Seq was performed on HBF and LBF clinical isolates grown in RPMI or RPMI supplemented with FCS at 90min, 4 and 24h. The HiSeq platform from Illumina provided an average of 24.4 million reads per sample. With the lowest sample (90 m_S39_3) having 13.6 million reads and the highest sample having 52.4 million reads (4h_S39_2) following Trimmomatic quality and adapter removal. The average GC of all reads was 39% and the average duplication rate per sample was 83%. All samples were deemed to have sufficient sequencing depth, greater than >10 million reads per sample, and were processed with Hisat2 with a 95% average alignment rate to the reference genome. All sequencing and alignment metrics are described in Supplementary Table 1.

We projected normalised DESeq2 gene counts into two dimensions that represented the largest distance between our samples. The dimensional reduction of multifactorial data, commonly referred to principal component analysis (PCA), allows for the differentiation of the samples with largest difference in its variables (PC1) and second largest difference (PC2). We visualised the component spaces of the 1st three components. From the first component it was possible to see that there was little variance between our 3 replicates per sample and that the largest separation, i.e. the *x* axis PC1 representing 34% of the explained variance, was due to difference in time variable of biofilm formation primarily between early (90min and 4h) and late-stage (24h) biofilm formation (Supplementary Fig. 2A). Visualising the ordination of the 1st and 3rd components revealed larger separation of the components according to the two media over the 3rd component (Supplementary Fig. 2B).

As an initial comparison we investigated any drug response related transcripts that could differentiate between our high and low biofilm forming clinical isolates. When comparing the upregulated genes through over-representation analysis of GO pathways in HBF compared to LBF we observe a number of drug related pathways. To achieve this we compared the 24h biofilm of HBF in RPMI only to the 24h biofilm of LBF in RPMI only. The over-represented pathways included drug metabolic process and a number of transmembrane transport terms in the Biological Process and Molecular Function related ontologies (Supplementary 3A). Drug transporters play an important role in drug resistance and there exists two superfamilies of exporters in *Candida* species, the ATP-binding cassette (ABC) and Major Facilitator superfamily (MFS) [43]. Through the CGD we were able to screen the genome revealing a total of 57 drug transporter, efflux or other drug resistance related genes [44]. We Identified all known ABC and MFS transporters in addition to putatively and predicted drug resistance proteins. The expression was then compared between HBF and LBF in all of our conditions. The expression patterns between our different times and conditions were then clustered hierarchy to identify any HBF specific expression of drug resistance. From our observations there was not a uniform regulation of drug transporters and drug resistance genes in either HBF or LBF. We did observe that the *Candida* drug resistance (*CDR*) family of genes *CDR1*, *CDR2*, *CDR3*, and *CDR11* were all upregulated in the HBF at the 24h. These are all previously identified or putatively identified members of the ABC family of transporters. Overexpressing isolates of the CDR family of genes have previously been shown to be related to fluconazole resistance as has the multidrug resistance (*MDR1*) gene that was overexpressed in the HBF in RPMI at 4h and 24h but not in the FCS [45,46]. The overexpression of specific ABC transporters at 24h may explain the increased resistance in HBF to fluconazole as described in Fig. 1. Similarly, the more varied regulation of other drug transporters and resistance mechanisms in LBF could relate to the lack of increased tolerance of HBF compared to LBF in response to polyenes and echinocandins.

We observed high levels of differentially expressed genes in both strains grown in RPMI compared to RPMI supplemented with FCS, with higher levels being observed in the HBF (Supplementary Fig. 4). Several common genes featured within the top down-regulated genes in FCS or up-regulated genes in RPMI (Supplementary Fig. 5). We observed that zinc transport was up-regulated in the RPMI only grown biofilms, and reciprocally *ZRT1/2* zinc transport genes and the *PRA1* zincophore gene was consistently down-regulated to a high level across each of our time points and biofilm conditions in FCS, with downregulation by Log_2_FC of ∼9 in LBF and ∼10 in HBF. The interaction between Pra and Zrt complexes allows for the efficient uptake of zinc in *C. albicans* [47]. Zinc is required by many enzymes in yeasts and is thought to be a key factor in *C. albicans* virulence and host-pathogen interactions [48]. This complex also been shown to be important for zinc homeostasis in regulating biofilm formation, confirmed in studies were conducted in the CA14 background [49]. Though this confirms our RPMI induced data, it is unclear how serum causes reciprocal effects.

**Fig. 4:**
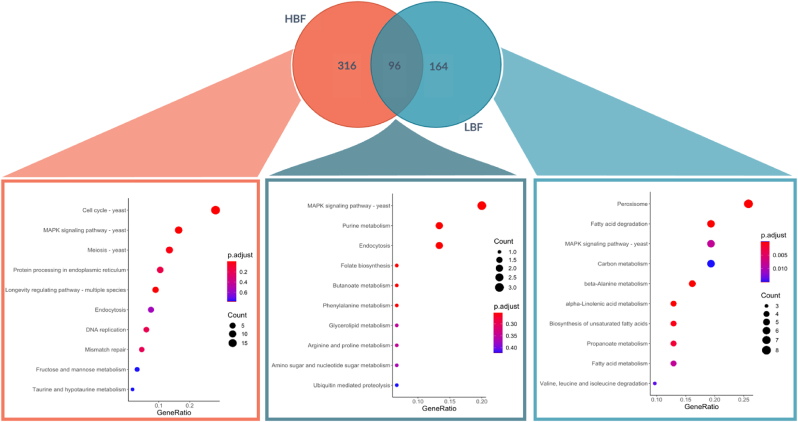
**Comparative analysis of the enriched KEGG terms serum in both low and high biofilm forming *C. albicans* isolates.** Differentially expressed genes that were upregulated in *C. albicans* isolates that were grown in serum compared to RPMI are compared. Those genes that were unique to HBF, LBF or overlapped between the two were submitted to over enrichment analysis against the known KEGG pathways for *C. albicans*.

In the presence of FCS upregulated genes for LBF and HBF were analysed for overlapping and unique genes. These data were overlayed as a Venn diagram and KEGG over representation analysis was performed to determine the enriched pathways (Fig. 4). We observed 316 genes that were uniquely upregulated in HBF, 96 upregulated in both phenotypes, and 164 unique to LBF. Only the LBF collection of genes achieved significantly overrepresented pathways according to our FDR corrected p value < 0.05. KEGG enrichment analysis of the LBF revealed fatty acid metabolism related genes *FOX2, FOX3, ECI1, CAT2, PEX11 and ANT1* were upregulated and led to the enrichment of the fatty acid metabolism related terms (Fig. 4 & Supplementary Figs. 5 and 6). The utilisation of non-sugar carbon sources, such as β-oxidation of fatty acids is known to be important for *C. albicans* virulence and nutritional flexibility. The fatty acid eicosanoid metabolites, such as prostaglandin E2 and thromboxane B2, present in serum have been shown to influence germ tube formation in *C. albicans* [50]*.* The fatty acid binding and acyl-coA related genes for acyl-coenzyme A oxidase *POX1* and *POX 1–3* and *PXP3* were specifically over-represented in LBF. β-oxidation of fatty acids in *C. albicans* takes place within the peroxisome. Acetyl units can then be integrated into the glyoxylate cycle or exported outside of the peroxisome by the carnitine shuttle system [51]. Members of the *ATO* gene family *ATO1*, *ATO10* and *ATO9* were over-represented in the term “plasma membrane acetate transport” and these are upregulated in the LBF forming strains in the presence of FCS. *ATO* genes are linked to acetate membrane transport and mutations in the *ATO* family have been demonstrated to effect the yeast to hyphal switch [52], and though these have never been described in classical biofilm studies, it is logical to imply their function given their role in morphogenesis. Moreover, their importance in metabolic adaptation reveals that the LBF phenotype may be more suited to stressful conditions than HBF isolates. Together, the data represents mechanisms by which LBF strains potentially utilise alternative carbon metabolic pathways from serum compounds, which in turn trigger yeast-hyphal morphogenesis and biofilm formation.

### 3.4 Metabolomics identified that arachidonic acid metabolism was important in biofilm induction

**Fig. 5:**
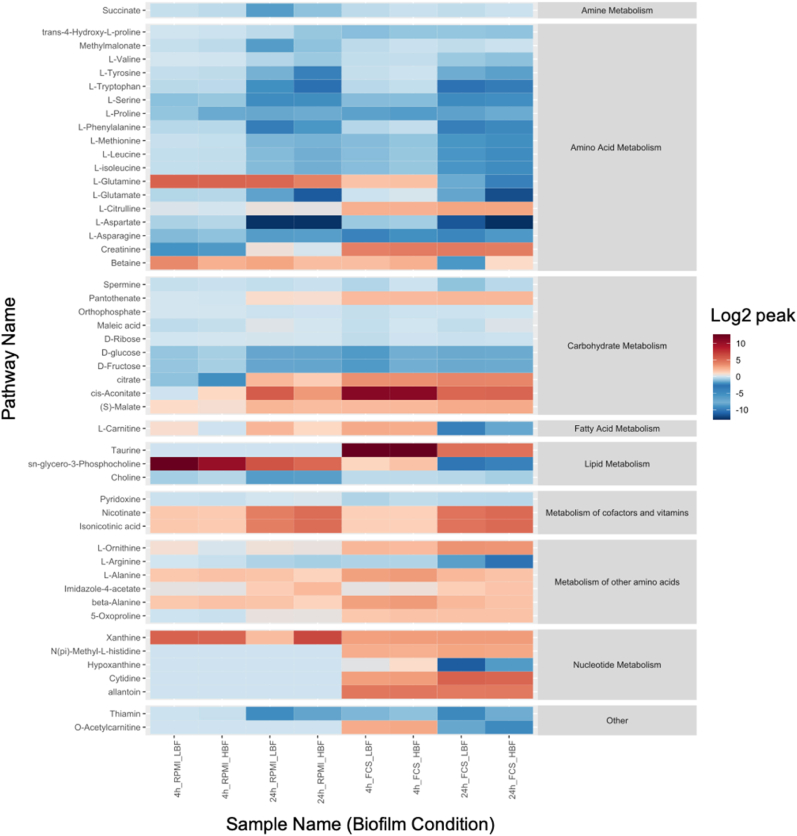
**Grid or heat plot of the relative abundance compared to the media control of the targeted or identified metabolites.** Positive log2 values infer secretion of metabolites to the media and negative values infer that metabolites have been spent from the media. Values are log2 metabolites relative abundance which have been divided by the control values. The values have also been grouped into their super-pathway metabolic groups according to their KEGG classification.

Based upon our previous observations that heterogeneity in clinical isolates was highly dependent on metabolic adaptation, metabolomics was performed on phenotypically distinct clinical isolates grown in the presence or absence of serum for 4h and 24h [18]. The metabolites were detected by both targeted and untargeted LCMS. Targeted analysis based upon known standards with accurate mass and retention time successfully identified 49 compounds within the samples tested. There were numerous differences within the list of standard compounds when *C. albicans* was grown in FCS supplemented RPMI compared to RPMI alone, and here major and significant changes in amino acids were found. *C. albicans* utilised betaine, L-glutamine, L-asparagine, L-isoleucine and L-leucine in FCS over the incubation time. Whereas in the RPMI only media L-tryptophan and creatinine were utilised (Fig. 5 & Supplementary Figs. 7–8). Amino acid sensing and uptake in *C. albicans* is a known inducer of hyphal morphogenesis [53]. The amino acid sensor Ssy1p (Csy1p) is nutritional response sensor that activates the amino acid permease *AAP* genes [54]. The SPS plasma membrane sensor, comprised of the subunits Ssy1, Pr32 and Ssy5, activated in fungi upon sensing of extracellular amino acids results in proteolytic cleavage of the transcription factor Stp2 that controls amino acid permeases [55]. Spt2 has now been shown to have a central effect in biofilm formation, leading to a global metabolic imbalance, suggesting it could act as a key target alongside antifungal therapy [56]. In addition to biofilm formation, amino acid metabolism is associated with virulence and triggering of yeast to hyphae morphogenesis [57,58]. Several amino acids were differentially detected in FCS in both LBFs and HBFs (p < 0.05), including L-arginine, L-alanine, beta-alanine, S-oxyproline and L-carnitine (Fig. 5 & Supplementary Figs. 7–9). These amino acids are related to several KEGG pathways within *C. albicans*. Amino acids are thought to trigger signalling pathways in *C. albicans*, either directly or in directly, such as arginine in the activation of the SPS plasma sensor and/or indirectly through their biosynthesis leading to alkalinisation [59]. Similar to our observation that amino acids were spent in the media supplemented with FCS, it has also been observed that arginine metabolic processes play an important role in type strain studies utilising hyphal deficient knockouts, within which they suggest a role for the yck2 in the regulation of arginine metabolism and the ROS response of *C. albicans* [60]. It has also been observed that significant changes in the metabolic response to the environment are present in *C. albicans* SC5314. Gallo et al. (2022) observed shifts in proline, arginine aspartate and glutamate amino acid uptake and catabolism in response to the hyphal growth condition of M199 media. These findings are consistent with our findings that there is increased intracellular uptake of amino acids in hyphal and biofilm inducing conditions [61].

**Fig. 6:**
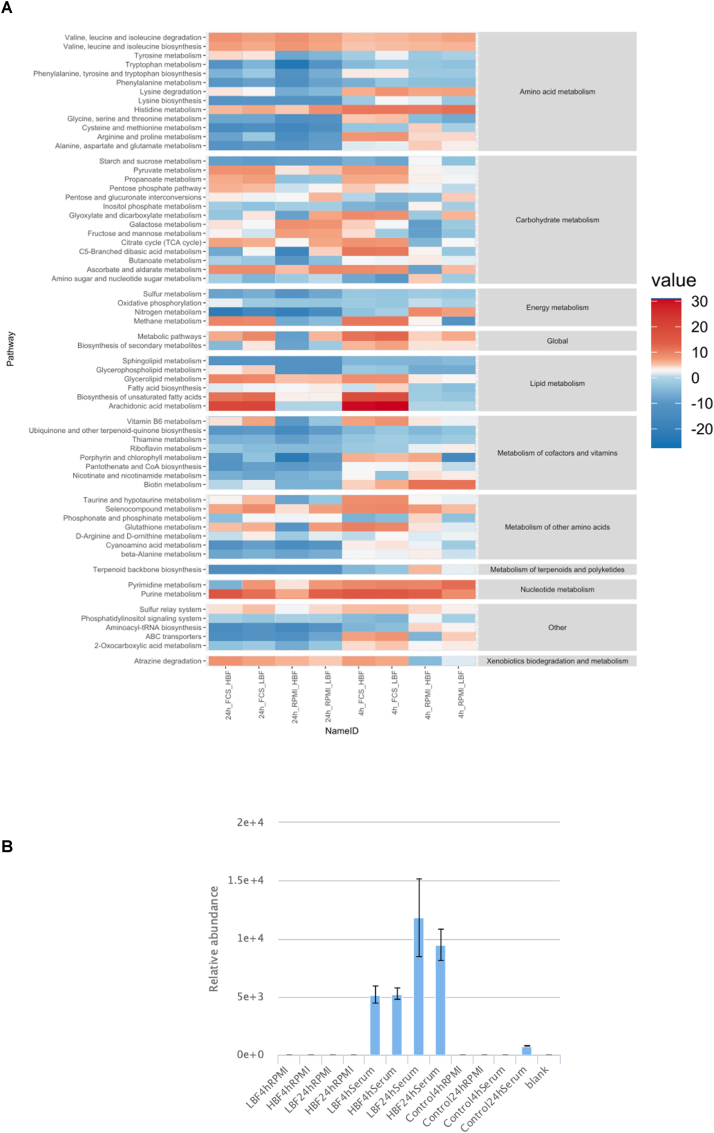
**Grid plot of the level of pathway activation according to Pathway Activity Profiling (PAPi).** Summary of activity by relative square root transformed activity score. The grid plot contains pathways found to be significant between any of our sample groups by ANOVA followed by Benjamini-Hochberg FDR. Positive or negative values indicate secretion to or utilisation from the media **(A)**. A bar chart of arachidonic acid displays relative abundance in each biofilm time-point and media combination acquired by LC-MS **(B)**.

Differentially enriched pathways were determined using the PAPi algorithm, which accounts for changes in both spent and secreted metabolomic changes. Significantly changing pathways with a corrected p-value of ≤0.05 according to ANOVA are shown in Fig. 6. These pathways were then further filtered to identify those that specifically changed between RPMI and RPMI + serum (Supplementary Figs. 10 and 11). Introduction of the serum resulted in a reprogramming within many of the metabolic super pathways. The starkest differences being observed in the lipid metabolic pathways. Arachidonic acid (AA) metabolism was the most significantly regulated pathway due to a high abundance of metabolite measured when our isolates were grown in the presence of FCS, and no activity of the metabolites related to this pathway in our isolates grown in RPMI only. It was found to be significant at both time points in both groups of strains (p = 4.37e-13). However, AA is not produced by *C. albicans*, so it is likely cleaving arachidonic acid from phospholipids found within the FCS. This would additionally explain the lack of AA in the RPMI controls.

Biosynthesis of unsaturated fatty acids and the related propanoate metabolic pathway were highly enriched in RPMI plus FCS compared to the RPMI alone putatively assigned compounds arachidonic acid, docosahexadonic acid, oleic acid and steraic acid. Previously it has been identified that AA and oleic acid play an important role in the synthesis of prostaglandins, precursors of prostaglandin E2 (PGE2) [62]. Production of prostaglandins by *C. albicans* has been shown to modulate hyphal formation and control the host inflammatory response [50]. Similarly, it has been shown that inhibition of cyclogeneses' Cox1p and Cox2p, which mediate the transition of AA to prostaglandin, effectively limits yeast-hyphal morphogenesis and limits biofilm formation [63]. AA metabolite relative abundance was visualised as a bar chart due to our PAPi findings. It was found to be a very serum specific metabolite, with low levels or not detected in the RPMI only or serum deficient cultures (Fig. 6A and B). Glycerolipid metabolism had a higher positive activity score in all FCS samples compared to RPMI which was deemed to be significant at all time points (p = 4.37e-13). A positive activity score (AS) is indicative of metabolites from this pathway being secreted into the media by *C. albicans*. Glycerolipid intermediates were secreted in the FCS-containing cultures and glycerophospholipids were more spent in RPMI only cultures. These pathways were putatively annotated by their intermediate's glycerol, glycerone, D-glycerate and D-glyceraldehyde.

Overall, the metabolic footprint of clinical isolates grown in serum supplemented media varies markedly compared to RPMI only. These results demonstrate the metabolic flexibility and plasticity undertaken by *C. albicans* in response to nutrient stress. They additionally highlight the complex differences in biofilm heterogeneity in response to nutrient stress between seemingly phenotypically distinct strains.

### 3.5 Arachidonic acid induces biofilm formation in a low biofilm phenotype

**Fig. 7:**
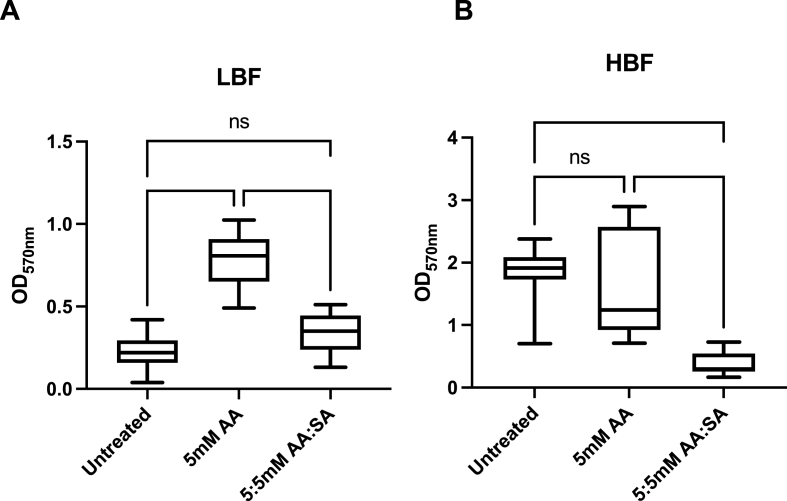
**Promotion of Biofilm formation in *C. albicans* clinical isolates by Arachidonic Acid**. Biofilms of **(A)** HBF (n = 5) and **(B)** LBF (n = 5) *C. albicans* isolates were grown for 24h before being treated with arachidonic acid (AA) only or AA combined with the COX pathway inhibitor, salicylic acid (SA). All compounds were administered at 5 mM and incubated with the biofilms for a further 24h. Following incubation, biofilm biomass was quantified by crystal violet staining. The absorbance of the bound dye was measured at 570 nm (****; *P* < 0.0001).

Increased levels of AA were detected within the spent media from biofilms grown in FCS, potentially derived from phospholipids. We hypothesised that this would activate the AA cascade leading to prostaglandin production which has previously been demonstrated to influence *Candida* morphogenesis. We tested the hypothesis that AA was important in *C. albicans* biofilm formation and pathogenesis. To achieve this, we treated biofilms with 5 mM AA on its own and in combination with the cyclooxygenase (COX) pathway inhibitor salicylic acid (Fig. 7). Salicylic acid is a widely used COX pathway inhibitor and has been shown to inhibit *Candida* biofilms through inhibition of prostaglandin PGE_2_ [63,64]. Not only this, but PGE_2_ released from *C. albicans* has been shown to influence the growth of bacteria such as *S. aureus* within mixed biofilms [65]. We observed that the addition of AA to our growth media had a significant influence on the biofilm formation of our 5 LBF strains providing an almost 3-fold increase in mean biomass. The HBF strain response was not significant despite our analyses showing a similar trend of higher AA within the spent media of HBF strains. These strains already record particularly high absorbance with the crystal violet assay, and this may be due to HBF strains already saturating this particular assay.

We observed that the effect of biofilm induction in the LBF was simultaneously ameliorated with the addition of our COX pathway inhibitor. Similarly, in the HBF strains there was a significant reduction in biofilm formation with the addition of salicylic acid. These findings indicate that the AA pathway is important in the biofilm induction that is observed in the LBF strains in the presence of serum. It also suggests that LBF clinical isolates can modulate their biofilm formation through the use of host derived nutrients.

### 3.6 Integrated analysis reveals fatty acid and amino acid metabolism plasticity

**Fig. 8:**
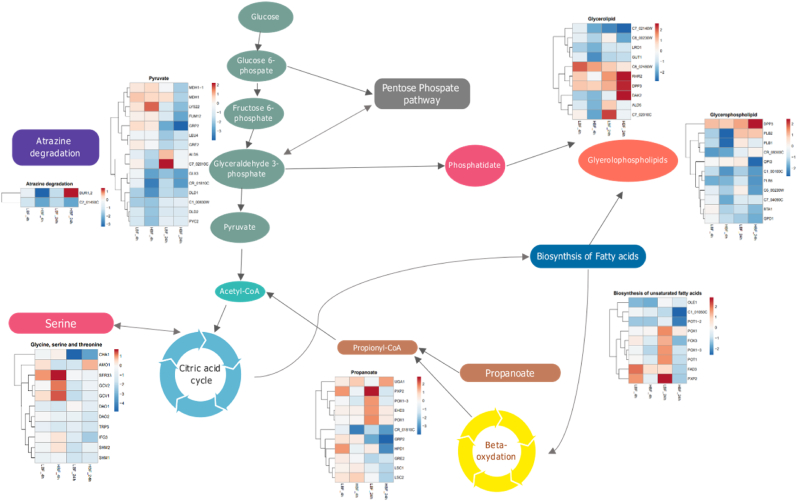
**Differential expression of genes associated with key metabolic pathways.** Differential expression of *C. albicans* transcripts in FCS vs Serum was performed between LBF and HBF at 4h and 24h. Those genes that were differentially expressed and that also belonged to a metabolic pathway. These pathways were determined to be significant by PAPi analysis based upon differences in activity score between our sample groups according to FDR corrected ANOVA. Pathways filtered first on significance within the PAPi analysis then pathways with significantly DE genes from at least one condition are displayed. This left genes involved in pathways that had a log_2_ fold change RPMI compared to serum for each strain at both 4 and 24h are represented within the heatmaps within each pathway shown.

We next aimed to provide further insight from our data sets which we have collected from *C. albicans* clinical isolates via integration. We therefore aimed to conceptually integrate our metabolomic and transcriptomic data sets. To investigate further we filtered our list of activated pathways, as determined by PAPi, to those that contained significantly up or down regulated genes in FCS compared to RPMI only cultures according to our cut-offs of log_2_FC > 1.5 and adjusted p-values (<0.05). We found several pathways with significantly up- or down-regulated genes in the presence of serum in both the HBF and LBF isolates. Overlaid pathways with log_2_FC of transcript abundance in Fig. 8 illustrates activated pathways with overlapping gene expression.

Taken together the metabolomic and transcriptomic data demonstrate the highly flexible reprogramming of metabolic pathways in *C. albicans* isolates in response to serum. *C. albicans* derives nutrients from fatty acids, lipids, and proteins in addition to the traditional glucose carbon source [57]. *C. albicans* is known to utilise a modified oxidation pathway for the removal of propionyl-CoA derived from the degradation of fatty acids and amino acids [66].

*C. albicans* displays a high level of metabolic plasticity and flexibility and its ability to derive nutrient requirements in non-preferred carbon sources has been associated with its virulence within the host [67]. The response in LBF isolates is similar to the transcriptional response observed in *C. albicans* in infected macrophages, where there is adaptation of the beta-oxidation and arginine metabolic pathways [68]. This adaptation has been linked to the yeast casein kinase 2 and is thought to regulate hyphal formation through the arginine degradation and beta-oxidation pathways that are observed in macrophage engulfment [60].The carbon metabolism plasticity and the utilisation of amino acids has been also hypothesised to sustain the growth of hyphae in diverse environments as well as provide a feedback mechanism for hyphal elongation [60,61]. Previously the *PXP2, FOX2, FOX3* and *POX1-3* genes involved in beta-oxidation were described in *C. tropicalis* and *C. parapsilosis* as significantly upregulated genes in response to blood [42]. Although the authors of the study observed that fatty acid metabolic response was not the same in *C. albicans.* However, we have observed phenotypic, metabolic, and transcriptional heterogeneity between clinical isolates.

## 4 Concluding remarks

Heterogeneity within *C. albicans* isolates illustrate the difficulty in describing specific response of fungal species that have strain to strain dependent responses to stress, nutrients, and the host. Survival strategies employed by *C. albicans* many and independently employed by different clinical isolates. The fatty acid β-oxidation pathway in *C. albicans* has been previously thought to be a non-essential component for virulence and survival [69]. However, LBF clinical isolates would appear to flexibly adapt to utilisation of this pathway in response to serum stimulation. Similar to an observed response in infection models, our LBF strains seem to utilise fatty acid metabolism during periods of nutrient stress. Lipid transporters are involved in the import and export of lipids from the cell [70], and are also thought to be important in *C. albicans* in biofilm formation and survival in response to stress and treatment. They are also signalling molecules that can activate biofilm formation, such as in the case of the farnesol and prostaglandins [62,71]. Notably, the phospholipase genes *PLB1* and *PLB2*, thought to be involved in host invasion, were both upregulated in serum in LBF. This points to host derived lipids and fatty acids within the serum serving as alternative or additional nutrient sources in *C. albicans.* Intermediates of these pathways could potentially be driving the morphogenesis and biofilm formation under these conditions.

Overall, these data highlight the importance of working with clinical strains and undertaking careful phenotypic characterisation in order to identify nuances within the molecular circuitry that may otherwise be overlooked by using standard laboratory strains. We have identified that the LBF phenotype can use arachidonic acid through this approach, a pathway that we can possibly exploit as a drug target.

## Transparency declarations

None to declare.

## CRediT authorship contribution statement

**Christopher Delaney:** Conceptualization, participated in study conception/design and experimental procedures and were responsible for preparation of the manuscript. **Bryn Short:** Conceptualization, participated in study, conception/design and experimental procedures and were responsible for preparation of the manuscript. **Ranjith Rajendran:** Conceptualization, participated in study conception/design. **Ryan Kean:** participated study design and preparation of the manuscript. **Karl Burgess:** participated study design and preparation of the manuscript. **Craig Williams:** participated study design and preparation of the manuscript. **Carol A. Munro:** participated study design and preparation of the manuscript. **Gordon Ramage:** conceived the study, participated in study design and data analysis and was responsible for producing the final manuscript. All authors contributed to the article and approved the submitted version.

## Author contributions

CD and BS participated in study conception/design and experimental procedures and were responsible for preparation of the manuscript. RR contributed to study conception/design. RK, KB, CW, and CM participated study design and preparation of the manuscript. GR conceived the study, participated in study design and data analysis and was responsible for producing the final manuscript. All authors contributed to the article and approved the submitted version.

## Declaration of competing interest

The authors declare the following financial interests/personal relationships which may be considered as potential competing interests: Prof Ramage is an Editor for Biofilm. He has played no role in the review or editorial process for this article.

## Data Availability

Data will be made available on request.
