# Supplementary material for: An integrated transcriptomic and metabolomic approach to investigate the heterogeneous Candida albicans biofilm phenotype
Source: Biofilm. 2023 Mar 12;5:100112. doi: 10.1016/j.bioflm.2023.100112 (PMC10034394; doi:10.1016/j.bioflm.2023.100112)
Supplement: Multimedia component 1 [file mmc1.pptx]

## Slide 1
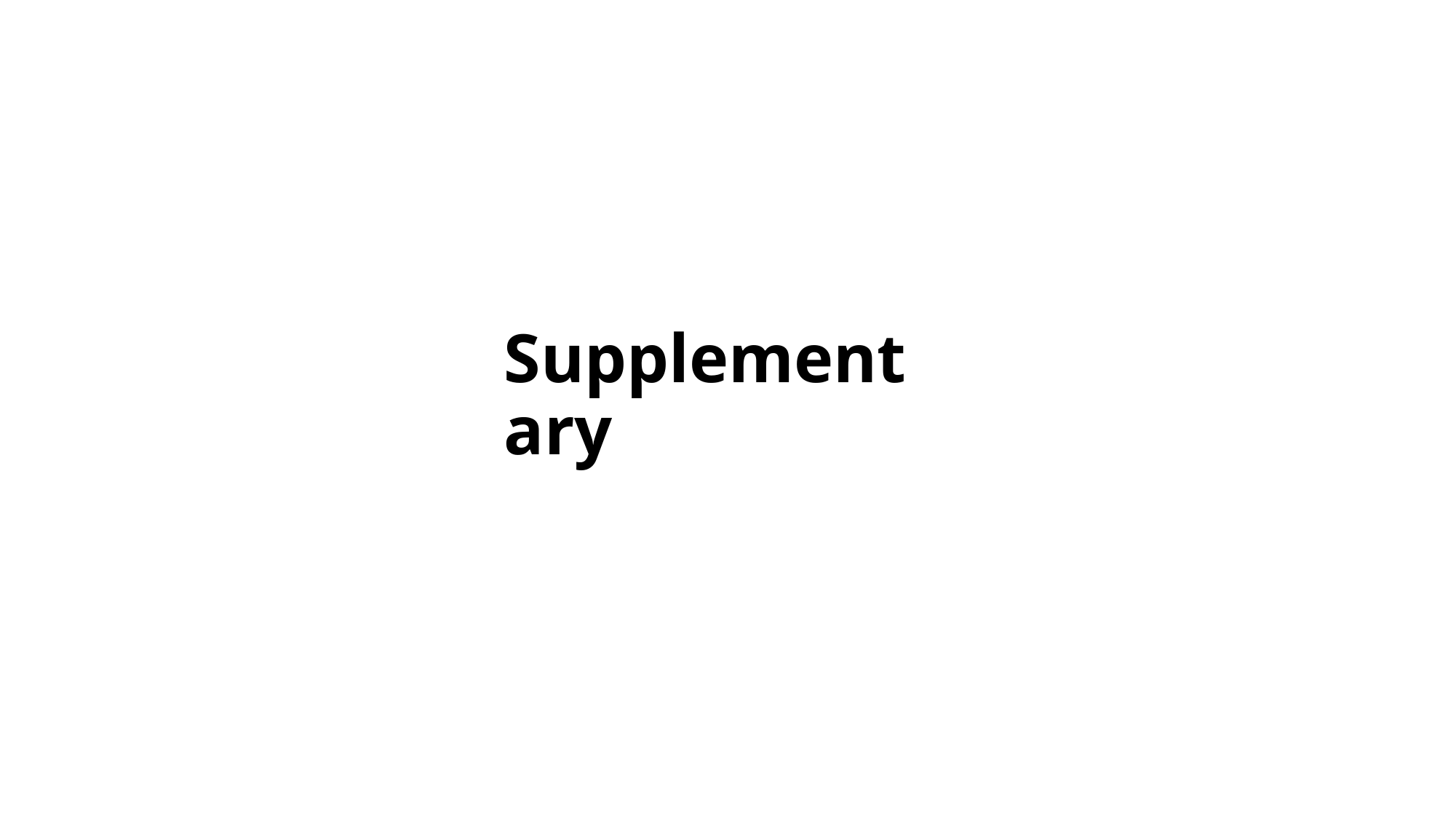

# Supplementary

## Slide 2
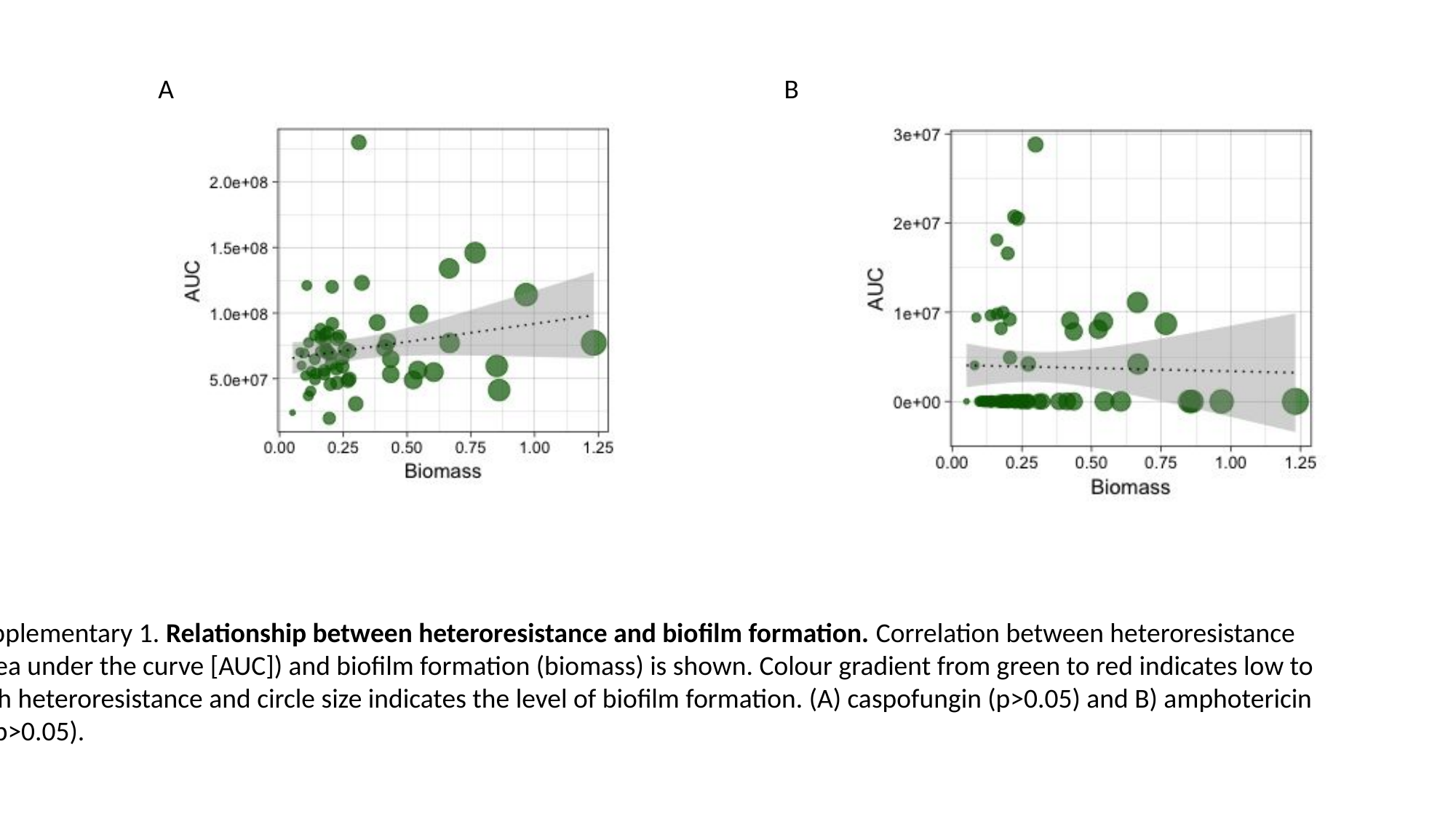

A
B
Supplementary 1. Relationship between heteroresistance and biofilm formation. Correlation between heteroresistance (area under the curve [AUC]) and biofilm formation (biomass) is shown. Colour gradient from green to red indicates low to high heteroresistance and circle size indicates the level of biofilm formation. (A) caspofungin (p>0.05) and B) amphotericin B (p>0.05).

## Slide 3
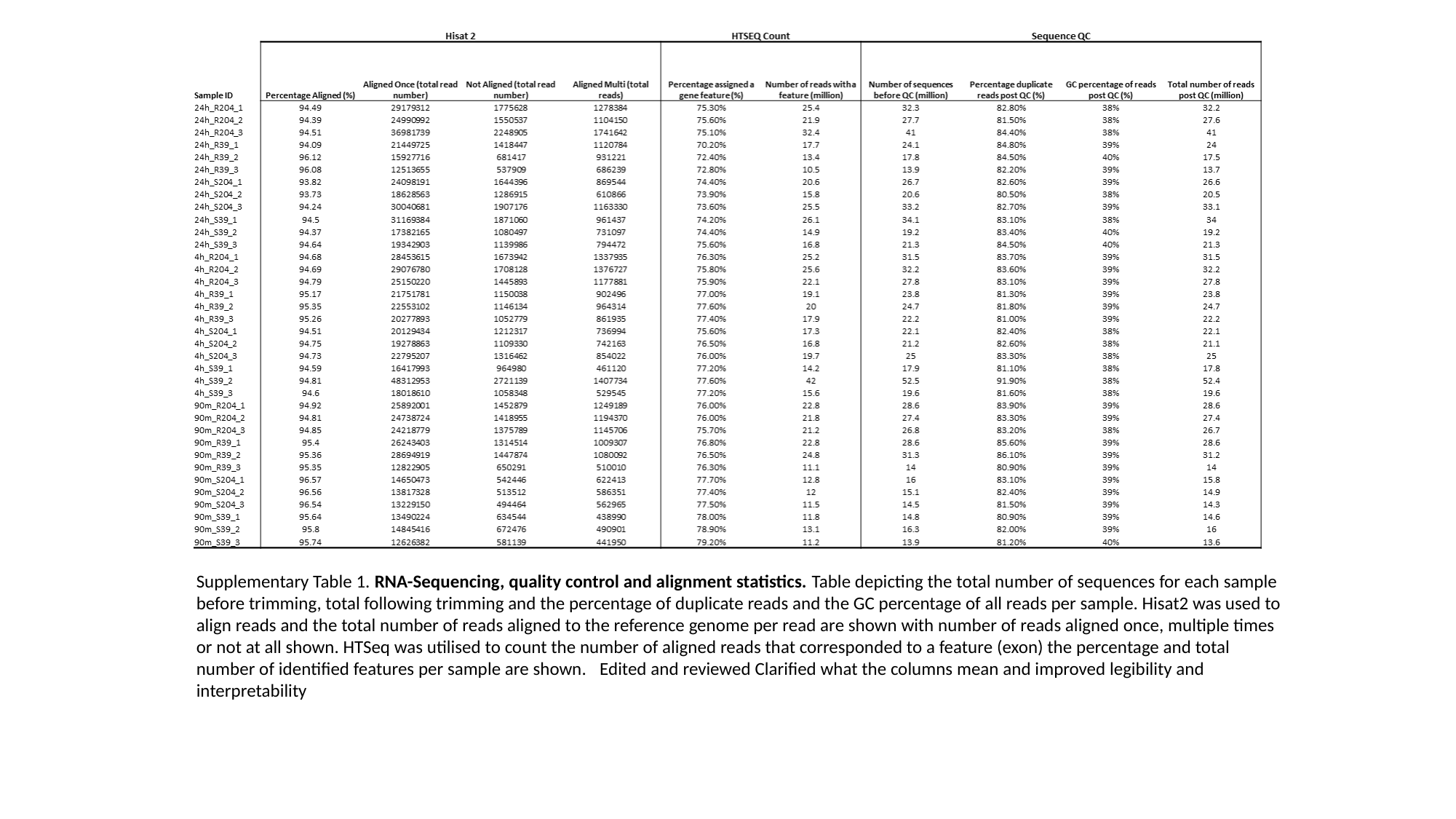

Supplementary Table 1. RNA-Sequencing, quality control and alignment statistics. Table depicting the total number of sequences for each sample before trimming, total following trimming and the percentage of duplicate reads and the GC percentage of all reads per sample. Hisat2 was used to align reads and the total number of reads aligned to the reference genome per read are shown with number of reads aligned once, multiple times or not at all shown. HTSeq was utilised to count the number of aligned reads that corresponded to a feature (exon) the percentage and total number of identified features per sample are shown.   Edited and reviewed Clarified what the columns mean and improved legibility and interpretability

## Slide 4
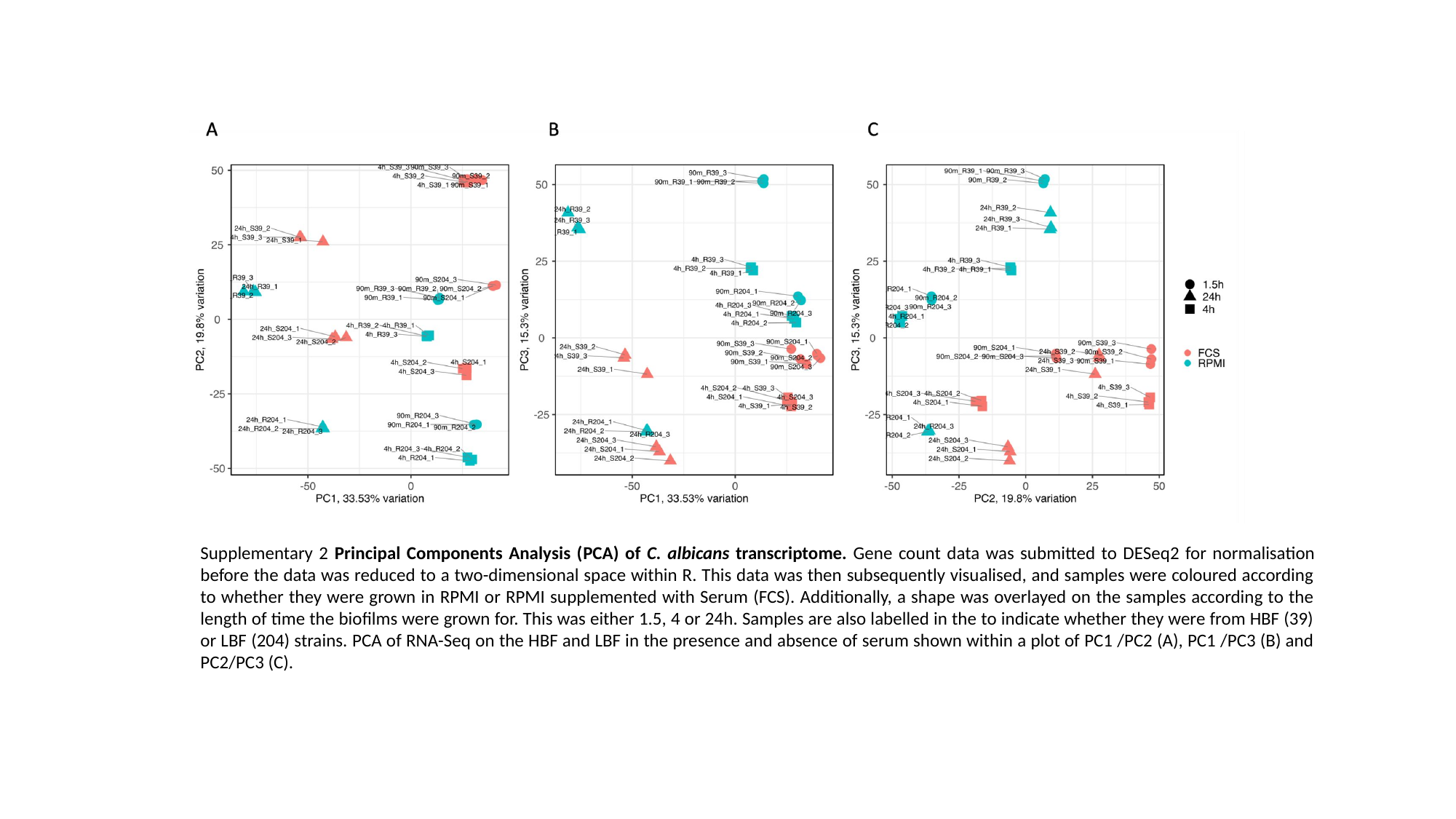

Supplementary 2 Principal Components Analysis (PCA) of C. albicans transcriptome. Gene count data was submitted to DESeq2 for normalisation before the data was reduced to a two-dimensional space within R. This data was then subsequently visualised, and samples were coloured according to whether they were grown in RPMI or RPMI supplemented with Serum (FCS). Additionally, a shape was overlayed on the samples according to the length of time the biofilms were grown for. This was either 1.5, 4 or 24h. Samples are also labelled in the to indicate whether they were from HBF (39) or LBF (204) strains. PCA of RNA-Seq on the HBF and LBF in the presence and absence of serum shown within a plot of PC1 /PC2 (A), PC1 /PC3 (B) and PC2/PC3 (C).

## Slide 5
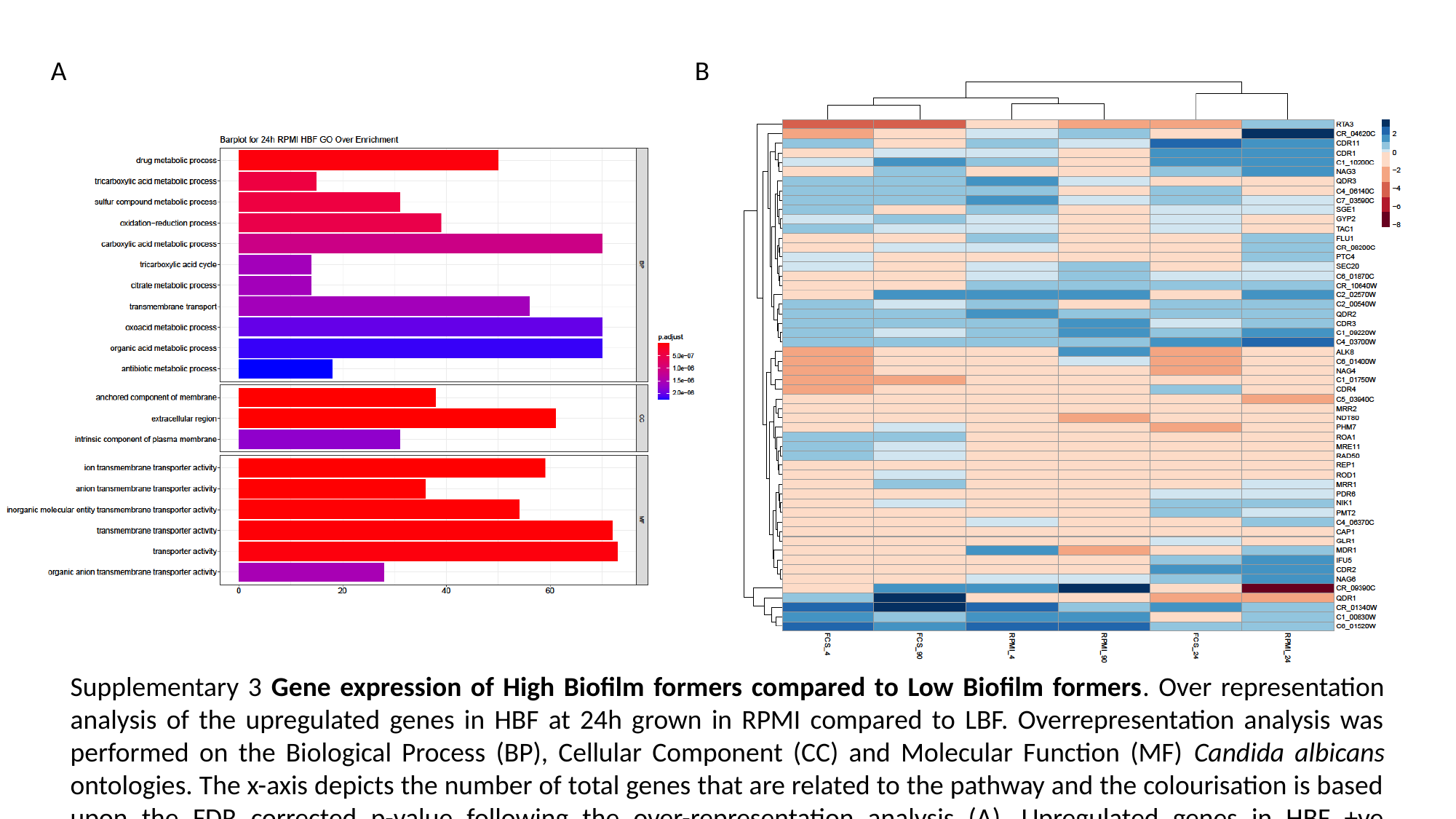

A
B
B
Supplementary 3 Gene expression of High Biofilm formers compared to Low Biofilm formers. Over representation analysis of the upregulated genes in HBF at 24h grown in RPMI compared to LBF. Overrepresentation analysis was performed on the Biological Process (BP), Cellular Component (CC) and Molecular Function (MF) Candida albicans ontologies. The x-axis depicts the number of total genes that are related to the pathway and the colourisation is based upon the FDR corrected p-value following the over-representation analysis (A). Upregulated genes in HBF +ve (colourised in blue) and LBF –ve (colourised in red) are depicted in a heatmap and clustered hierarchically. The variables depicted are the expression of genes HBF compared to LBF in RPMI(+/-FCS) at either 90 min, 4h or 24h.

## Slide 6
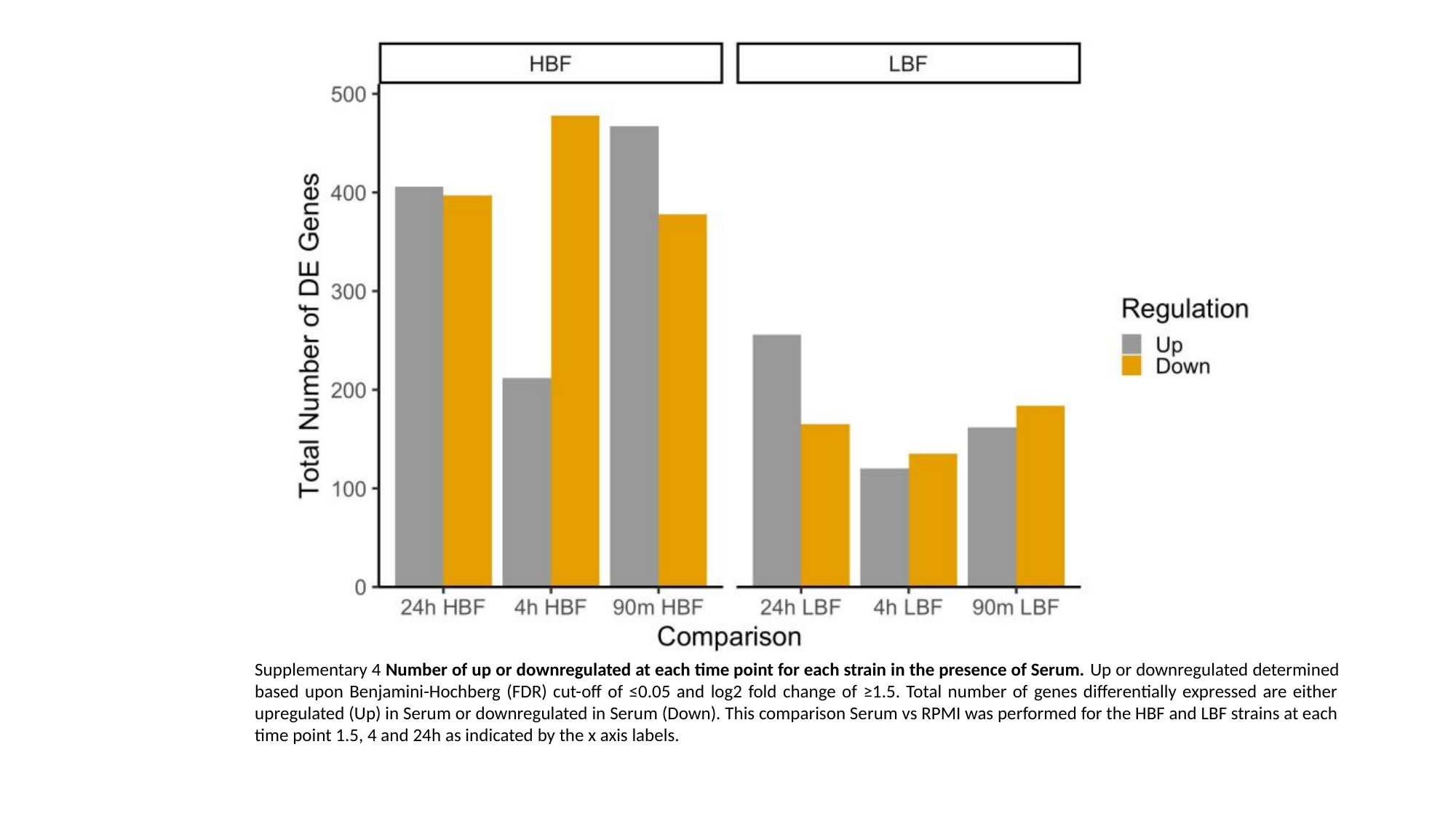

Supplementary 4 Number of up or downregulated at each time point for each strain in the presence of Serum. Up or downregulated determined based upon Benjamini-Hochberg (FDR) cut-off of ≤0.05 and log2 fold change of ≥1.5. Total number of genes differentially expressed are either upregulated (Up) in Serum or downregulated in Serum (Down). This comparison Serum vs RPMI was performed for the HBF and LBF strains at each time point 1.5, 4 and 24h as indicated by the x axis labels.

## Slide 7
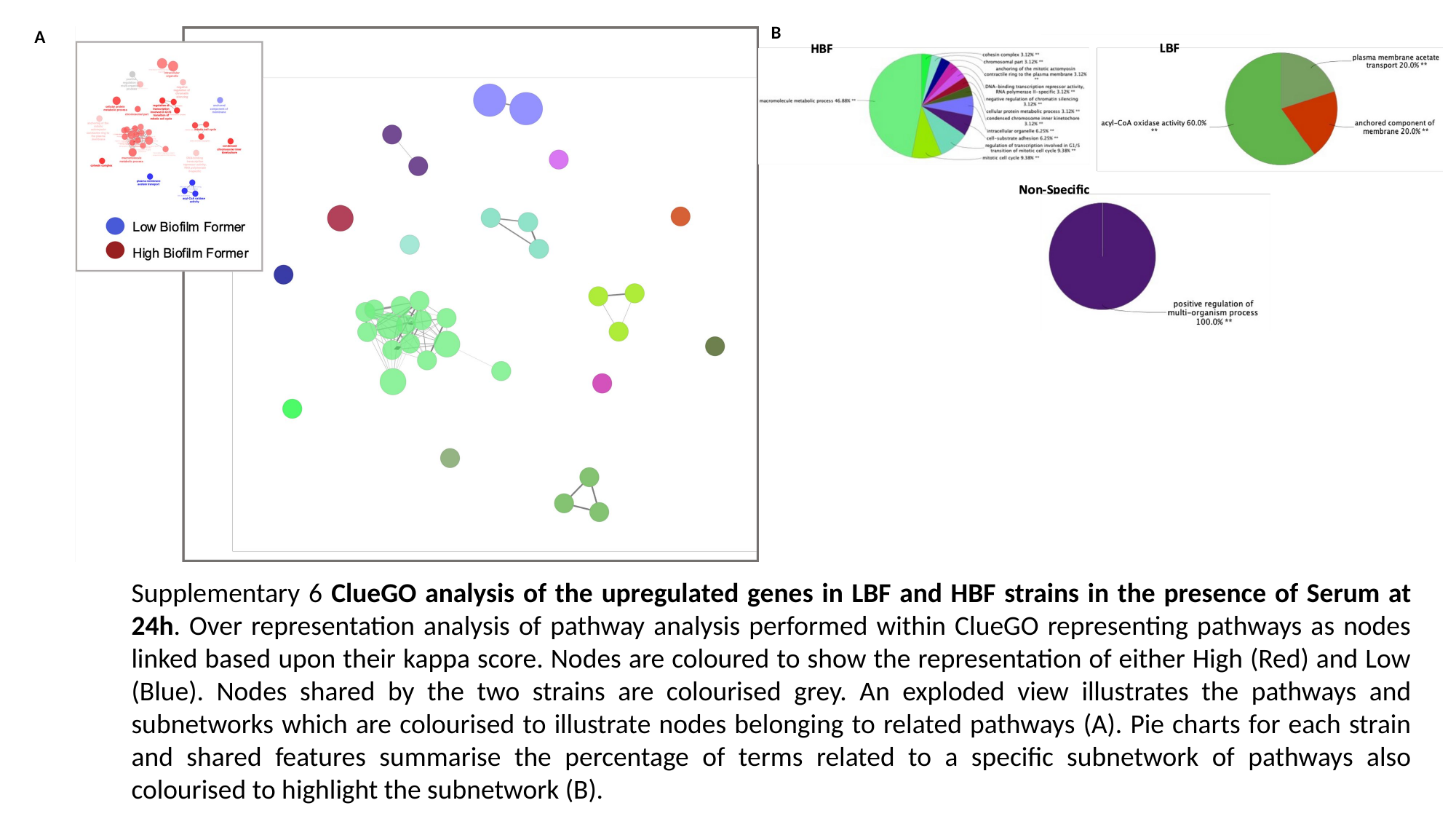

B
A
Supplementary 6 ClueGO analysis of the upregulated genes in LBF and HBF strains in the presence of Serum at 24h. Over representation analysis of pathway analysis performed within ClueGO representing pathways as nodes linked based upon their kappa score. Nodes are coloured to show the representation of either High (Red) and Low (Blue). Nodes shared by the two strains are colourised grey. An exploded view illustrates the pathways and subnetworks which are colourised to illustrate nodes belonging to related pathways (A). Pie charts for each strain and shared features summarise the percentage of terms related to a specific subnetwork of pathways also colourised to highlight the subnetwork (B).

## Slide 8
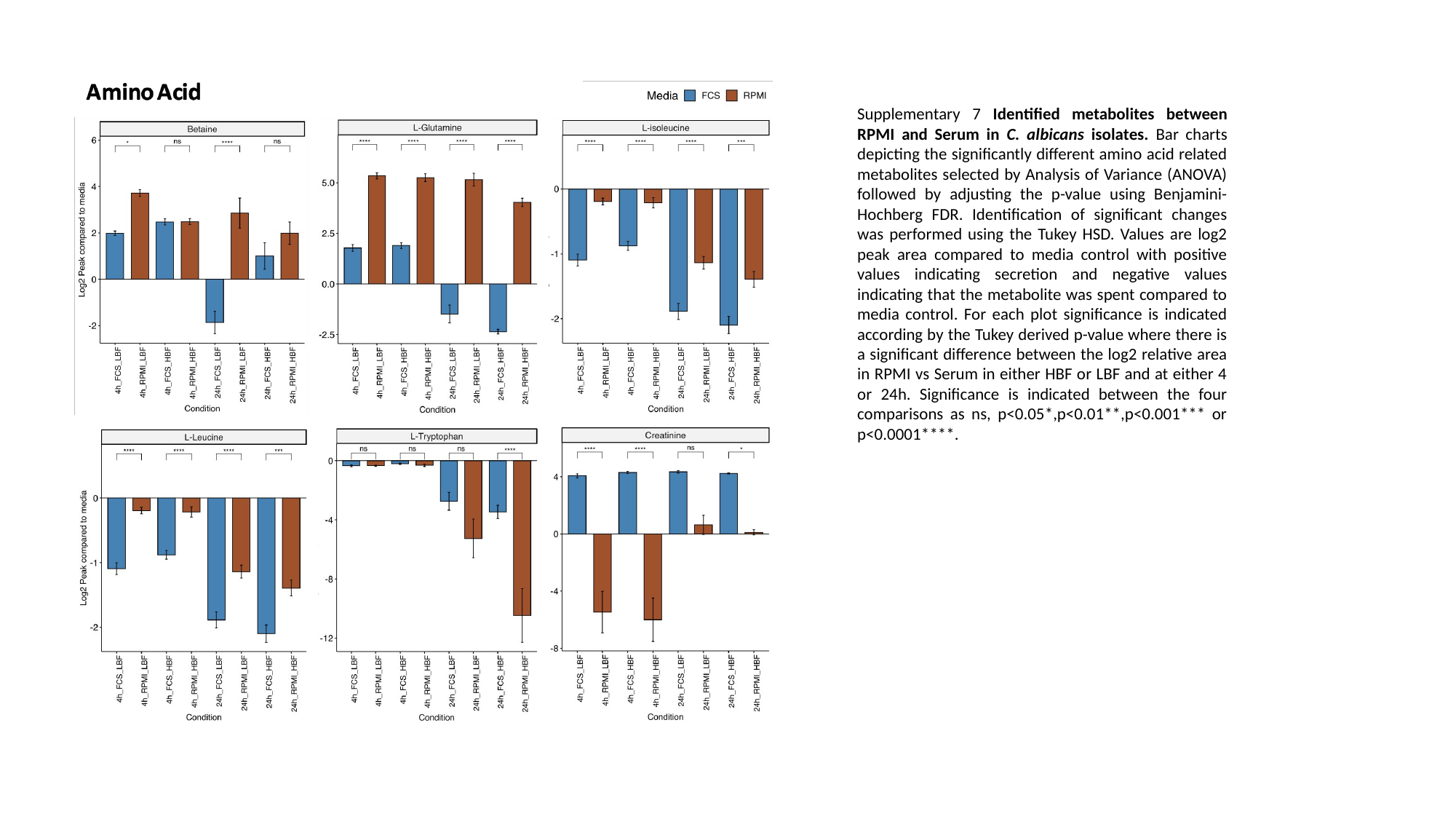

Supplementary 7 Identified metabolites between RPMI and Serum in C. albicans isolates. Bar charts depicting the significantly different amino acid related metabolites selected by Analysis of Variance (ANOVA) followed by adjusting the p-value using Benjamini-Hochberg FDR. Identification of significant changes was performed using the Tukey HSD. Values are log2 peak area compared to media control with positive values indicating secretion and negative values indicating that the metabolite was spent compared to media control. For each plot significance is indicated according by the Tukey derived p-value where there is a significant difference between the log2 relative area in RPMI vs Serum in either HBF or LBF and at either 4 or 24h. Significance is indicated between the four comparisons as ns, p<0.05*,p<0.01**,p<0.001*** or p<0.0001****.

## Slide 9
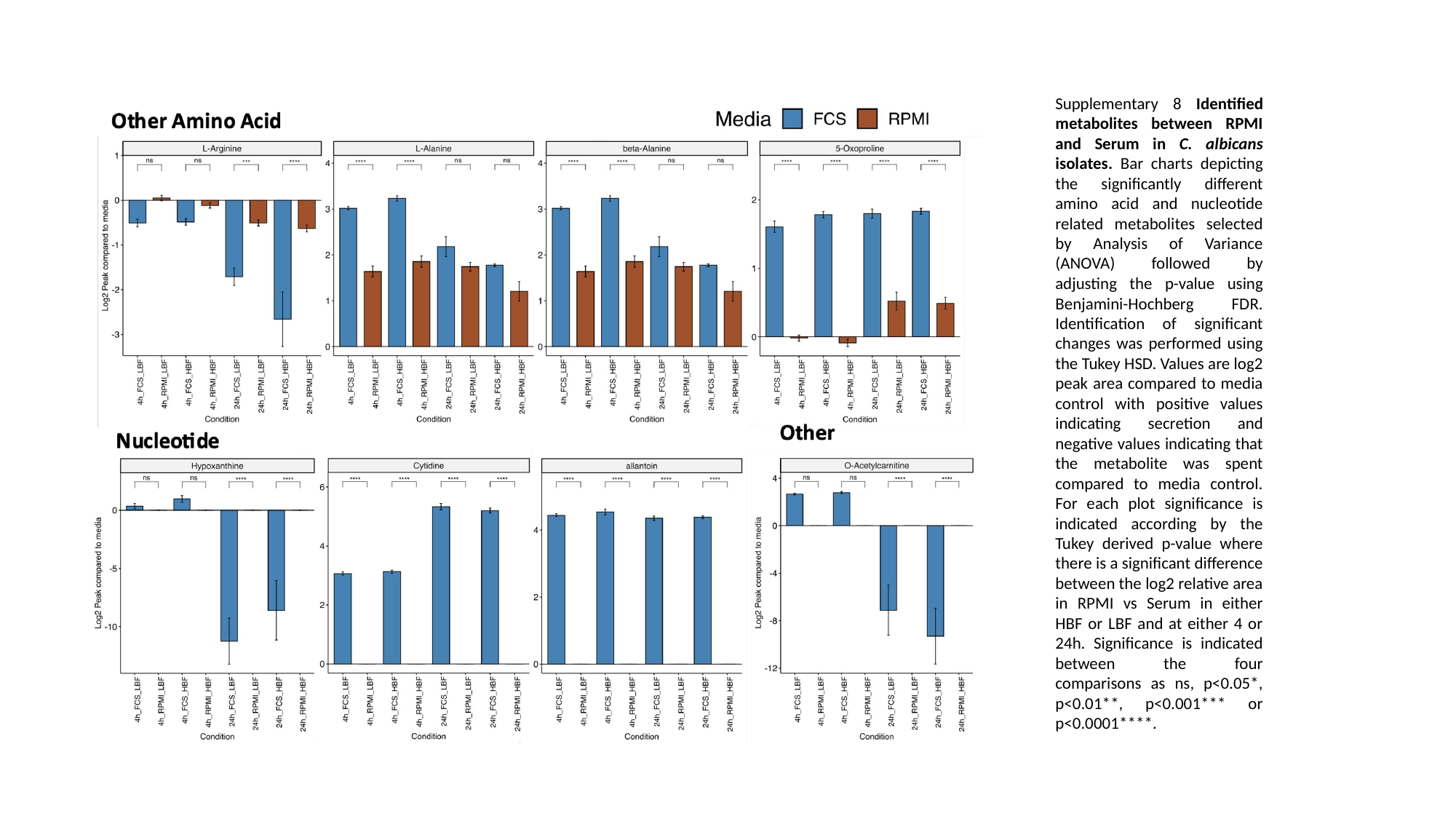

Supplementary 8 Identified metabolites between RPMI and Serum in C. albicans isolates. Bar charts depicting the significantly different amino acid and nucleotide related metabolites selected by Analysis of Variance (ANOVA) followed by adjusting the p-value using Benjamini-Hochberg FDR. Identification of significant changes was performed using the Tukey HSD. Values are log2 peak area compared to media control with positive values indicating secretion and negative values indicating that the metabolite was spent compared to media control. For each plot significance is indicated according by the Tukey derived p-value where there is a significant difference between the log2 relative area in RPMI vs Serum in either HBF or LBF and at either 4 or 24h. Significance is indicated between the four comparisons as ns, p<0.05*, p<0.01**, p<0.001*** or p<0.0001****.

## Slide 10
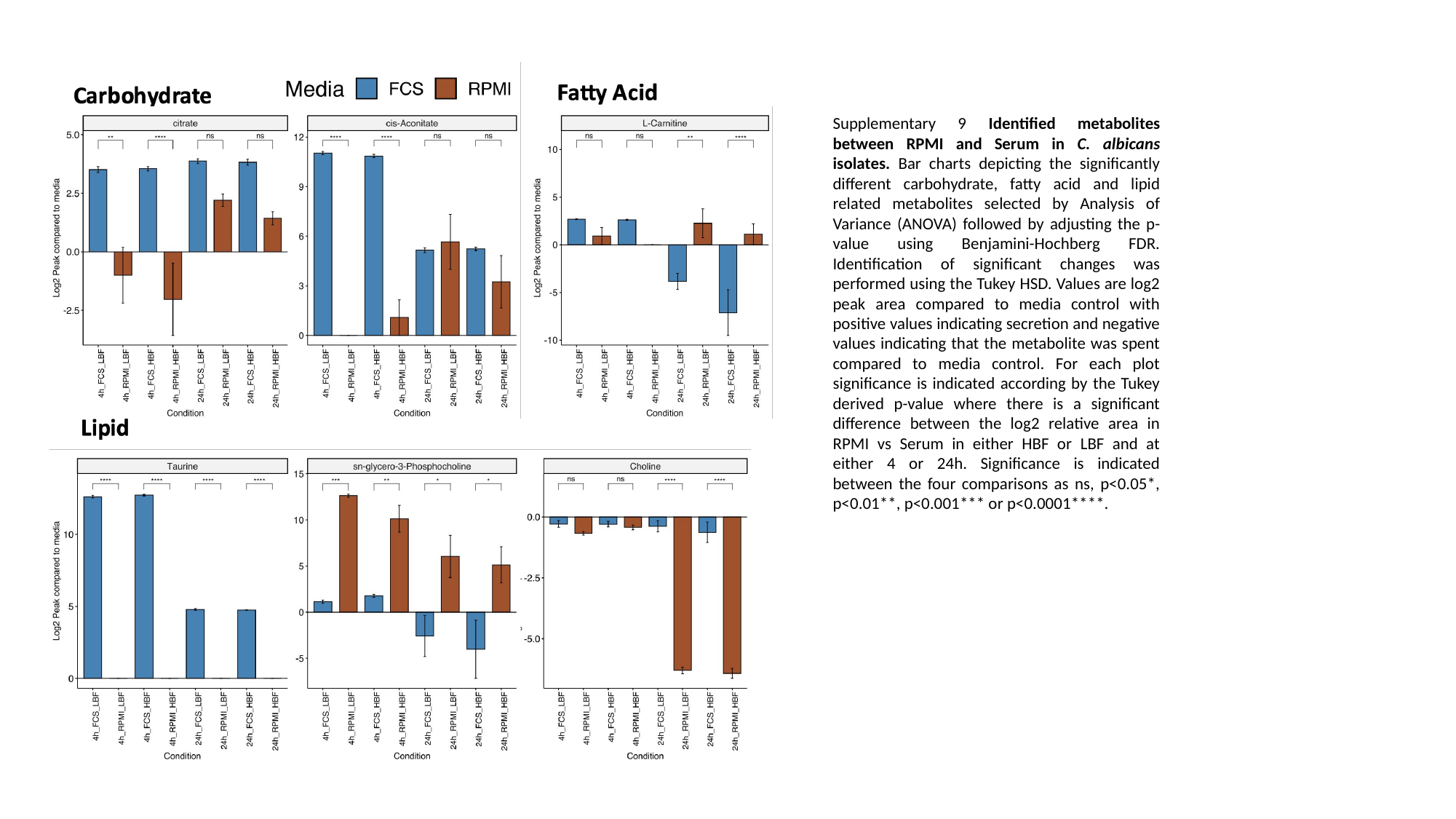

Supplementary 9 Identified metabolites between RPMI and Serum in C. albicans isolates. Bar charts depicting the significantly different carbohydrate, fatty acid and lipid related metabolites selected by Analysis of Variance (ANOVA) followed by adjusting the p-value using Benjamini-Hochberg FDR. Identification of significant changes was performed using the Tukey HSD. Values are log2 peak area compared to media control with positive values indicating secretion and negative values indicating that the metabolite was spent compared to media control. For each plot significance is indicated according by the Tukey derived p-value where there is a significant difference between the log2 relative area in RPMI vs Serum in either HBF or LBF and at either 4 or 24h. Significance is indicated between the four comparisons as ns, p<0.05*, p<0.01**, p<0.001*** or p<0.0001****.

## Slide 11
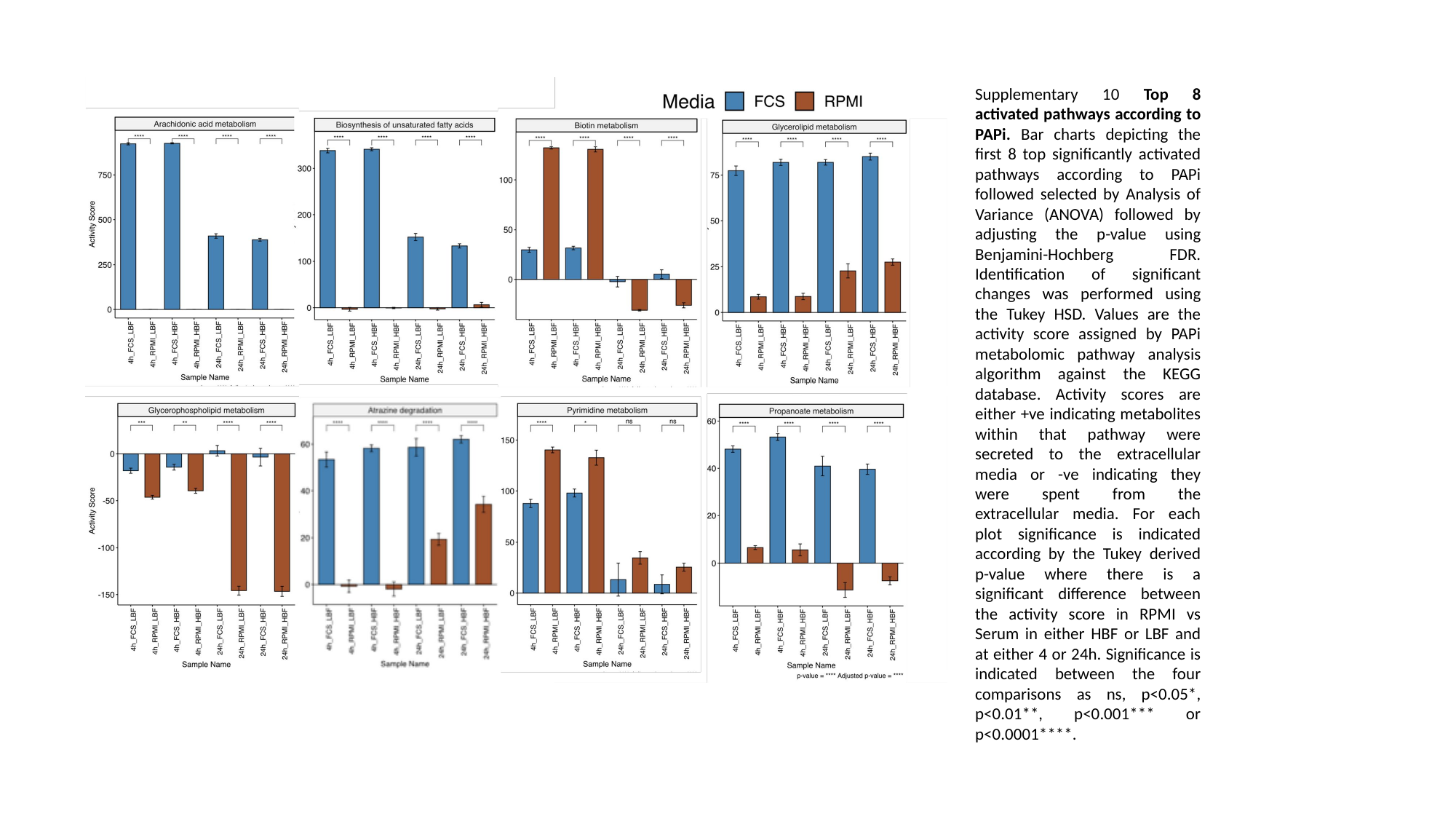

Supplementary 10 Top 8 activated pathways according to PAPi. Bar charts depicting the first 8 top significantly activated pathways according to PAPi followed selected by Analysis of Variance (ANOVA) followed by adjusting the p-value using Benjamini-Hochberg FDR. Identification of significant changes was performed using the Tukey HSD. Values are the activity score assigned by PAPi metabolomic pathway analysis algorithm against the KEGG database. Activity scores are either +ve indicating metabolites within that pathway were secreted to the extracellular media or -ve indicating they were spent from the extracellular media. For each plot significance is indicated according by the Tukey derived p-value where there is a significant difference between the activity score in RPMI vs Serum in either HBF or LBF and at either 4 or 24h. Significance is indicated between the four comparisons as ns, p<0.05*, p<0.01**, p<0.001*** or p<0.0001****.

## Slide 12
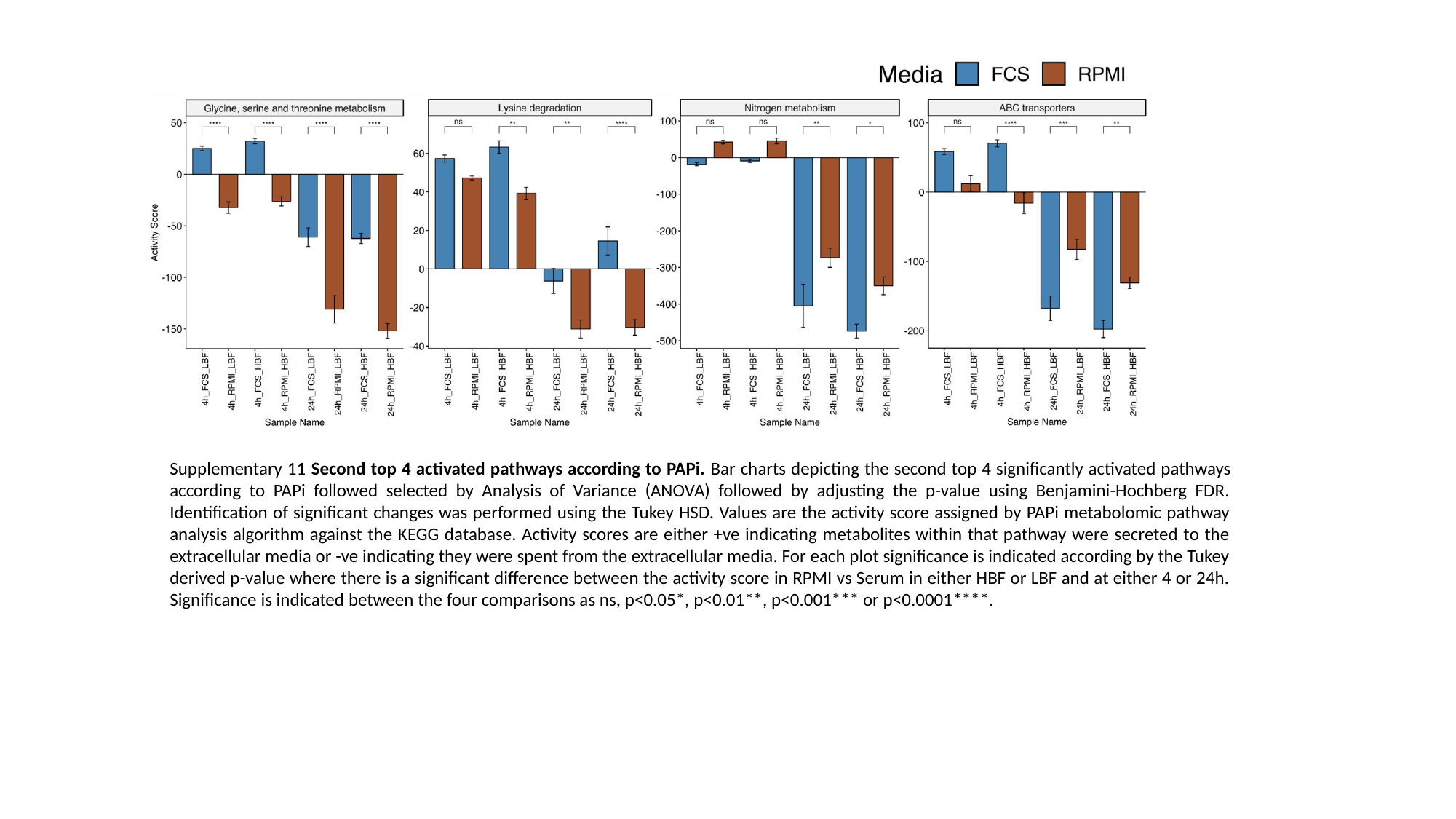

Supplementary 11 Second top 4 activated pathways according to PAPi. Bar charts depicting the second top 4 significantly activated pathways according to PAPi followed selected by Analysis of Variance (ANOVA) followed by adjusting the p-value using Benjamini-Hochberg FDR. Identification of significant changes was performed using the Tukey HSD. Values are the activity score assigned by PAPi metabolomic pathway analysis algorithm against the KEGG database. Activity scores are either +ve indicating metabolites within that pathway were secreted to the extracellular media or -ve indicating they were spent from the extracellular media. For each plot significance is indicated according by the Tukey derived p-value where there is a significant difference between the activity score in RPMI vs Serum in either HBF or LBF and at either 4 or 24h. Significance is indicated between the four comparisons as ns, p<0.05*, p<0.01**, p<0.001*** or p<0.0001****.

## Slide 13
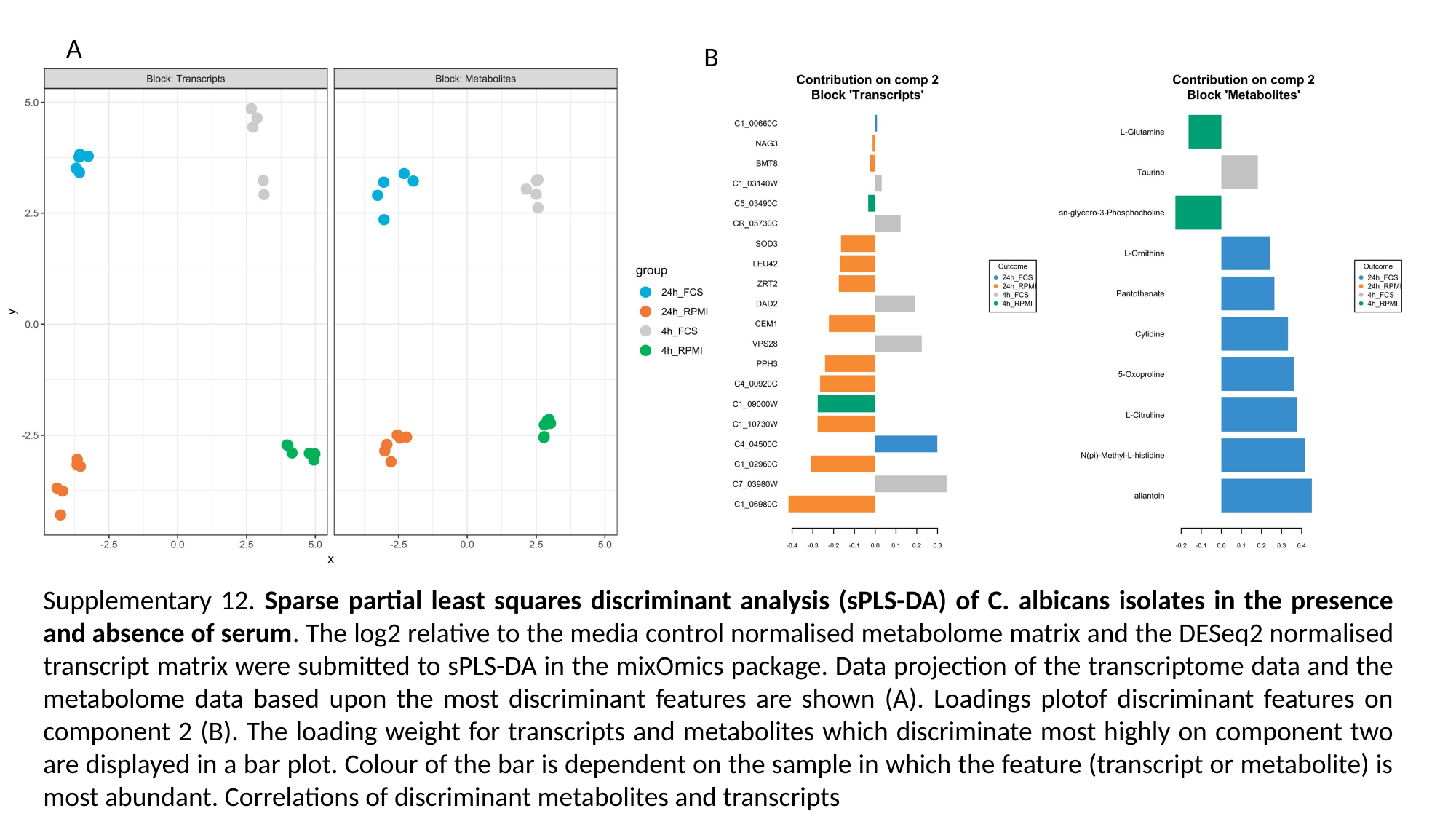

A
B
Supplementary 12. Sparse partial least squares discriminant analysis (sPLS-DA) of C. albicans isolates in the presence and absence of serum. The log2 relative to the media control normalised metabolome matrix and the DESeq2 normalised transcript matrix were submitted to sPLS-DA in the mixOmics package. Data projection of the transcriptome data and the metabolome data based upon the most discriminant features are shown (A). Loadings plotof discriminant features on component 2 (B). The loading weight for transcripts and metabolites which discriminate most highly on component two are displayed in a bar plot. Colour of the bar is dependent on the sample in which the feature (transcript or metabolite) is most abundant. Correlations of discriminant metabolites and transcripts
